# Semianalytical Treatment of Collective Vibrational Strong Coupling in Infrared Phononic and Plasmonic Nanoantennas

**DOI:** 10.1021/acs.jpcc.5c00458

**Published:** 2025-06-27

**Authors:** Jonathan Sepulveda, José Luis Montaño-Priede, Javier Aizpurua, Ruben Esteban

**Affiliations:** † Centro de Física de Materiales (CFM-MPC), CSIC-UPV/EHU, Paseo Manuel Lardizabal 5, Donostia-San Sebastián 20018, Spain; ‡ Department of Electricity and Electronics, FCT-ZTF, UPV/EHU, Leioa 48940, Spain; § Donostia International Physics Center (DIPC), Paseo Manuel Lardizabal 4, Donostia-San Sebastián 20018, Spain; ∥ IKERBASQUE, Basque Foundation for Science, Plaza Euskadi 5, Bilbao 48009, Spain

## Abstract

The interaction between nanoantenna electromagnetic modes and molecular vibrations enables key nanophotonic applications such as ultrasensitive vibrational spectroscopy and molecular sensing. Here, we develop a systematic analysis to obtain the collective coupling strength, *g*, between bright collective vibrational modes of an assembly of molecules and infrared modes of phononic and plasmonic nanoantennas. Our approach exploits the information in the electromagnetic fields as obtained from a single classical simulation, incorporated in semianalytical equations that enable to calculate *g* for any arbitrary molecular distribution, thus dramatically reducing the computational effort. These semianalytical equations are validated by comparing the results of the coupling strength with those obtained from a harmonic oscillator model by fitting the extinction cross-section spectra. Furthermore, we apply the semianalytical equations in our model to compare the values of *g* obtained for molecules surrounding both phononic and noble-metal plasmonic nanoantennas. Our results indicate that in the infrared, the coupling with noble-metal plasmonic nanoantennas achieves larger *g* than with phononic nanoantennas for any molecular distribution. However, due to the large plasmonic losses, reaching the strong coupling regime is more feasible in phononic nanoantennas. Additionally, we derive simplified analytical expressions of *g* valid when a nanoantenna is fully surrounded by molecules. These expressions reveal that the coupling strength is governed in this case by the ratio of the electromagnetic energy inside and outside the nanoantenna. Our results provide insights on the interaction of infrared nanoantennas with molecules at infrared frequencies, facilitating the design of optimized configurations for different applications in nanophotonics.

## Graphical abstract

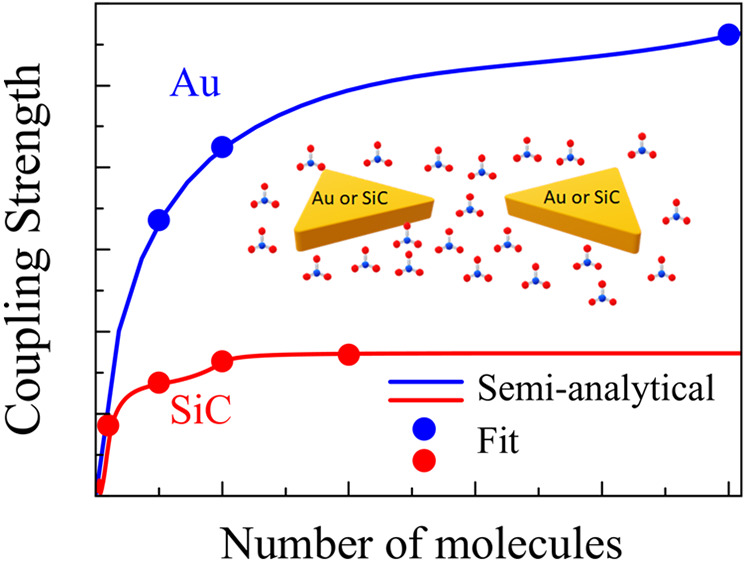

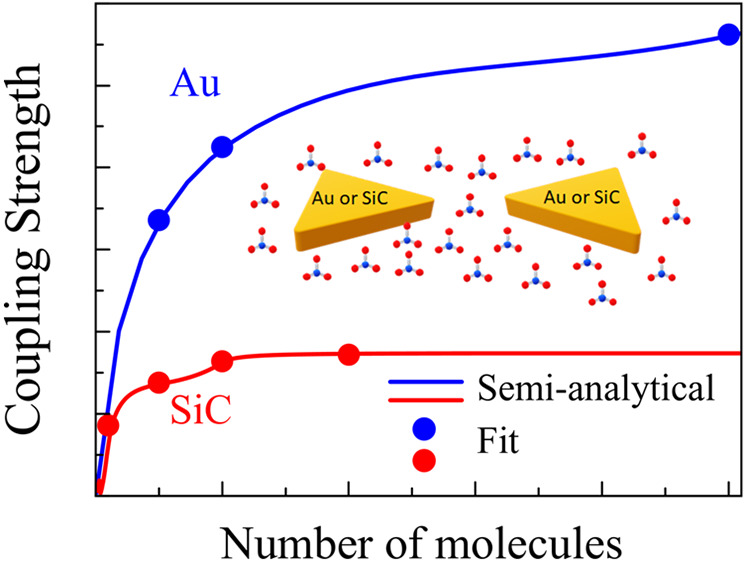

## 1 Introduction

The fabrication and use of nanoantennas to boost light-matter interaction at the nanoscale has been a central topic in nanophotonics^1,2^ since the extreme localization of the optical near fields in the proximity of the nanoantennas^3–6^ enables the control and manipulation of light well below the diffraction limit.^7^ When the near fields of an optical mode of such a nanoantenna interact with excitonic transitions of molecules or other quantum emitters (e.g., quantum dots or color centers in diamond), the emission properties of the emitters can be strongly modified due to the large density of states near the nanoantenna.^8–10^ Moreover, electromagnetic modes at different spectral ranges can also couple with and modify the properties of other dipolar excitations of matter, such as molecular vibrations at infrared frequencies.^11–13^

The interaction between a quantum emitter and a nanoantenna can be characterized by the coupling strength *g*. Depending on the ratio between *g* and the losses κ of the nanoantenna mode, we can identify two distinct interaction regimes: the weak coupling and the strong coupling regimes, each of them with its own characteristic phenomenology.^14–20^ In the weak coupling regime, the losses of the emitter excitations and nanoantenna mode are significantly larger than *g*, giving rise to an irreversible exchange of energy between the nanoantenna and the quantum emitter. In this situation, an increase in the spontaneous emission rate, known as the Purcell effect, can be observed.^21^ On the other hand, in the strong coupling regime, the losses are comparable or smaller than *g*, resulting in a coherent exchange of energy between the nanoantenna and the emitter (Rabi oscillations) before the energy is radiated to the far field.^16^ In this latter regime of interaction, the coupling results in the formation of new hybrid states, so-called polaritons.^20^ However, reaching strong coupling with a single quantum emitter is challenging, so that the optical modes of nanoantennas are often coupled in experiments with bright collective vibrational or excitonic excitations supported by ensembles of quantum emitters. This collective coupling enables much stronger coupling strengths between the emitters and the near field of the nanoantenna as compared to that for a single emitter.

The coupling with collective vibrational excitations supported in the infrared (IR) by ensembles of molecules has gained interest due to its potential application in ultrasensitive vibrational spectroscopy,^13,22,23^ and because of its potential to modify chemical reactions at the nanoscale.^24–28^ In this context, noble-metal^13,29–33^ and heavily doped semiconductor nanoantennas^34–36^ have been used to boost light-matter interaction, as they can strongly reduce the volume of the infrared mode,^7,13^ which increases the coupling strength and facilitates reaching the strong coupling regime. In this case, the nanoantenna supports localized surface plasmon polariton modes caused by the interaction of the electromagnetic field with the collective oscillation of the electrons in the metal or semiconductor.^37,38^ However, the tuning of plasmonic resonances in noble-metal nanoantennas into the infrared typically requires increasing the nanoantenna size up to dimensions of the order of micrometers, which limits miniaturization and enhances radiative losses^39,40^ detrimental to reaching the strong coupling regime.

An alternative approach to reach the strong coupling regime in the infrared is to couple collective vibrational excitations with localized surface phonon polariton modes of nanoantennas made of polar materials.^22,23,41–46^ These phononic nanoantennas also reduce very strongly the volume of the localized surface phonon polariton modes, in this case by exploiting the coupling of the electromagnetic field with the phonons of the material. Remarkably, phononic nanoantennas have smaller size and lower losses as compared to their noble-metal plasmonic counterpart,^41,42,46^ which makes them excellent candidates to reach the strong coupling regime with relatively few molecules.^23,46^

In this work, we systematically analyze the collective coupling strength *g* between bright collective vibrational modes involving many molecules and IR modes of bowtie nanoantennas. Furthermore, we compare the coupling of the collective excitations with phononic and with IR gold plasmonic nanoantennas, to understand the advantages and disadvantages of these two types of nanostructures. We perform the analysis with the use of two methods. First, we use a typical coupled harmonic oscillator model to obtain *g* by fitting the simulated spectra of the nanoantenna-molecule system. This methodology is widely used by the community of nanophotonics^18,45,47–50^ but requires a new simulation and fit for each spatial distribution or number of molecules, making a systematic analysis challenging. Thus, we focus on a second approach based on semianalytical equations that only requires a single simulation, which serves to calculate *g* for all possible spatial distribution and number of molecules. This semianalytical approach is first implemented for weakly radiative (phononic) nanoantennas, and it is later extended to make it possible to analyze highly radiative (infrared plasmonic) nanoantennas. The semianalytical model also provides insights into the maximum coupling strength achievable when the nanoantennas are completely surrounded by molecules.

The article is organized as follows. In Section 2, we present the numerical methods used to simulate the response of the coupled system, together with the definitions of the different types of volumes considered. The coupling of phononic nanoantennas with molecules is considered in Sections 3.1–3.4. In Section 3.1, we introduce the molecules and the bowtie nanoantenna used in the simulations, and in Section 3.2, we obtain *g* by fitting the simulated spectra with the coupled harmonic oscillator model. The derivation of the semianalytical equation to obtain *g* systematically for any molecular distribution is detailed in Section 3.3, where we also apply this equation to the bowtie phononic nanoantenna. Additionally, in Section 3.4, we use the semianalytical equation to study how the coupling strength depends on the nanogap separation distance between the two prisms that form the bowtie nanoantenna. We discuss in detail the case where the nanoantenna is fully surrounded by molecules, which yields the maximum collective coupling strength attainable. The coupling of molecules with an IR gold plasmonic nanoantenna is analyzed in Sections 3.5–3.7, with the description of the gold bowtie plasmonic nanoantenna considered in Section 3.5. Then, in Section 3.6 we obtain *g* again by fitting the spectra of the system with the coupled harmonic oscillator model. Section 3.7 extends the systematic semianalytical methodology presented in Section 3.3 to the situation of strongly radiative plasmonic nanoantennas. Finally, in Section 3.8 we compare the values of *g* obtained in both phononic and noble-metal plasmonic bowtie nanoantennas, and we summarize the results and conclusions in Section 4.

## 2 Computational Methods and Volume Definitions

The classical response of the IR phononic (SiC) and plasmonic (gold) bowtie nanoantennas both uncoupled and coupled to the molecules is simulated using the software package Lumerical FDTD Solutions.^51^ When molecules are included in these simulations, they occupy a cubic region with side length *L*_mol_, where the center of this cubic region is located in the middle of the nanogap. This region occupies a volume $V_{mol}\approx L_{\mathrm{mol}}^{3}$ (V_mol_ is not exactly equal to $L_{\mathrm{mol}}^{3}$ because there are no molecules in the region occupied by the bowtie). To simulate the response under plane wave illumination, we use the total field scattered field (TFSF) source implemented in Lumerical (which considers pulsed illumination and afterward extracts the response at each individual frequency). The volume of the computational space is ≳(λ_max_/2)^3^, where λ_max_ is the maximum wavelength considered for the illumination pulse, which ensures that the perfectly matched layers do not affect the results for the SiC and gold plasmonic nanoantennas. In all simulations, we use a mesh accuracy of 8 to achieve high spatial resolution, along with finer meshes to better resolve the bowtie nanoantenna geometry, especially in the nanogap region (without conformal mesh refinement). We use absorbing boundary conditions with 30 perfectly matched layers at each boundary to minimize the reflections of the scattered fields. We save the electric and magnetic fields using 3D monitors, and obtain the scattering and absorption cross-section using the cross-section analysis group. In all simulations, the auto shut off is 10^–6^, which ensures that the electromagnetic fields fully propagate in the region of interest and avoids artificial ripples in the spectrum. We perform convergence testing to verify the reliability of our results.

We note additionally, that, in the analysis of coupling strength below, we introduce different types of volumes. To make the discussion easier to follow, we introduce next a brief definition of each of them:

- V_mol_: Volume occupied by the ensemble of molecules. In the calculations, we consider that the molecules occupy either a cubic volume centered at the middle of the nanogap, or a region selected to maximize the coupling strength for a fixed number of molecules. In the latter case, the molecules are placed progressively in the regions with the strongest near fields, continuing this process until the entire computational volume is filled.
- V_int_: Cubic volume of integration centered at the middle of the nanogap. When analyzing the coupling of the SiC phononic nanoantenna with molecules fully surrounded it, we also divide this volume explicitly into the volume inside (V_SiC_) and outside (V_mol_) the nanoantenna.
- V_μ_: Effective volume occupied by one molecule.
- V_qst_: Volume resulting from the direct integration of the electromagnetic energy density of a nanoantenna mode, for any V_int_. V_qst_ is particularly useful to analyze the coupling with small (quasistatic) nanoantennas.
-
$V_{\mathrm{qst}}^{\mathrm{eff}}$ : Effective mode volume obtained from the dependence of V_qst_ on V_int_. $V_{\mathrm{qst}}^{\mathrm{eff}}$ is found to be accurate for small nanoantennas.
- Ṽ_rdc_: Complex volume resulting from integrating the square of the radiation-subtracted electromagnetic fields following quasi-normal mode theory. This value is appropriate for weakly and strongly radiative nanoantennas
-
${Ṽ}_{\mathrm{rdc}}^{\mathrm{eff}}$ : Complex effective mode volume obtained from the V_int_ → ∞ limit of Ṽ _rdc_. $\left|{Ṽ}_{\mathrm{rdc}}^{\mathrm{eff}}\right|$ is equivalent to $V_{\mathrm{qst}}^{\mathrm{eff}}$ for small nanoantennas, and it is also well suited to analyze strongly radiative nanoantennas.
- V_gap_: Estimated volume of the nanogap region, which we define in terms of the size of the tip of the bowtie and of the nanogap separation distance. This volume is useful to give a more intuitive picture of the different effective volumes and of the physical volume occupied by the ensemble of molecules.

## 3 Results and Discussion

### 3.1 Coupling with Phononic Nanoantennas

We first consider the coupling between molecular vibrations of many molecules and a phononic mode of a nanoantenna. Figure 1a shows a schematic representation of the system, a bowtie nanoantenna composed of two triangular prisms separated by a nanogap and surrounded by molecules. This configuration is chosen because it shows a region of strong fields (i.e., a hot electromagnetic spot) in the nanogap, which enables efficient coupling between the nanoantenna modes and the molecular vibrations that can be controlled by selecting the portion of molecules located inside and outside the hotspot. Additionally, this architecture is relatively easy to fabricate compared to other geometries^52,53^ and has often been used in previous experimental,^4,54,55^ and theoretical work.^44,56,57^ The white region in the sketch represents the strong field enhancement in the nanogap and the nanoantenna dimensions are shown in Figure 1b. Each triangular prism of the bowtie is 455 nm long, 511 nm wide, and *t* = 75 nm thick, and the three corners are rounded with a radius of *r* = 30 nm. We vary the nanogap distance *d*_g_ between the two prisms to control the strength and confinement of the induced fields. The system is excited by a plane wave of electric field amplitude E_0_ polarized along the bowtie axis (*x*-axis), which propagates in the -*z* direction normal to the top surface of the nanoantenna (see coordinate axis in Figure 1b). The bowtie is made of silicon carbide (SiC) and is placed in vacuum. The dielectric function of SiC is modeled using a Lorentzian function according to^44,58^

1 $$\varepsilon_{\mathrm{SiC}}\left(\omega \right)=\varepsilon_{0}\varepsilon_{\mathrm{SiC},\infty }\left[1+\frac{\left(\omega_{l}^{2}-\omega_{t}^{2}\right)}{\omega_{t}^{2}-\omega^{2}-i\omega \gamma_{\mathrm{SiC}}}\right]$$

where ω_*l*_ and ω_*t*_ are the longitudinal and transverse phononic (angular) frequencies, respectively, and γ_SiC_ is the damping rate (due to absorption losses), with **ℏ** *ω*_*l*_ = 0.12 eV, **ℏ** *ω*_*t*_ = 0.098 eV, *ℏγ*_SiC_ = 0.59 meV, **ℏ** the reduced Planck constant, *ε*_mol,∞_ = 6.7 the high-frequency permittivity, and *ε*_0_ the vacuum permittivity. Equation 1 corresponds to a simplified description of the dielectric function of SiC that does not consider anisotropy, which is usually small in SiC polytypes.^58,59^ We plot in Figure 1c the real (blue solid line) and imaginary (red dashed line) parts of the dielectric function as a function of photon energy (bottom axis). For easier reference, we indicate the corresponding wavenumber in cm^–1^ in the upper axis. The real part of the dielectric function is negative between the longitudinal and transverse phononic frequencies, so that localized surface phononic modes can be excited in this region of the spectrum (called Reststrahlen band).

**Figure 1:**
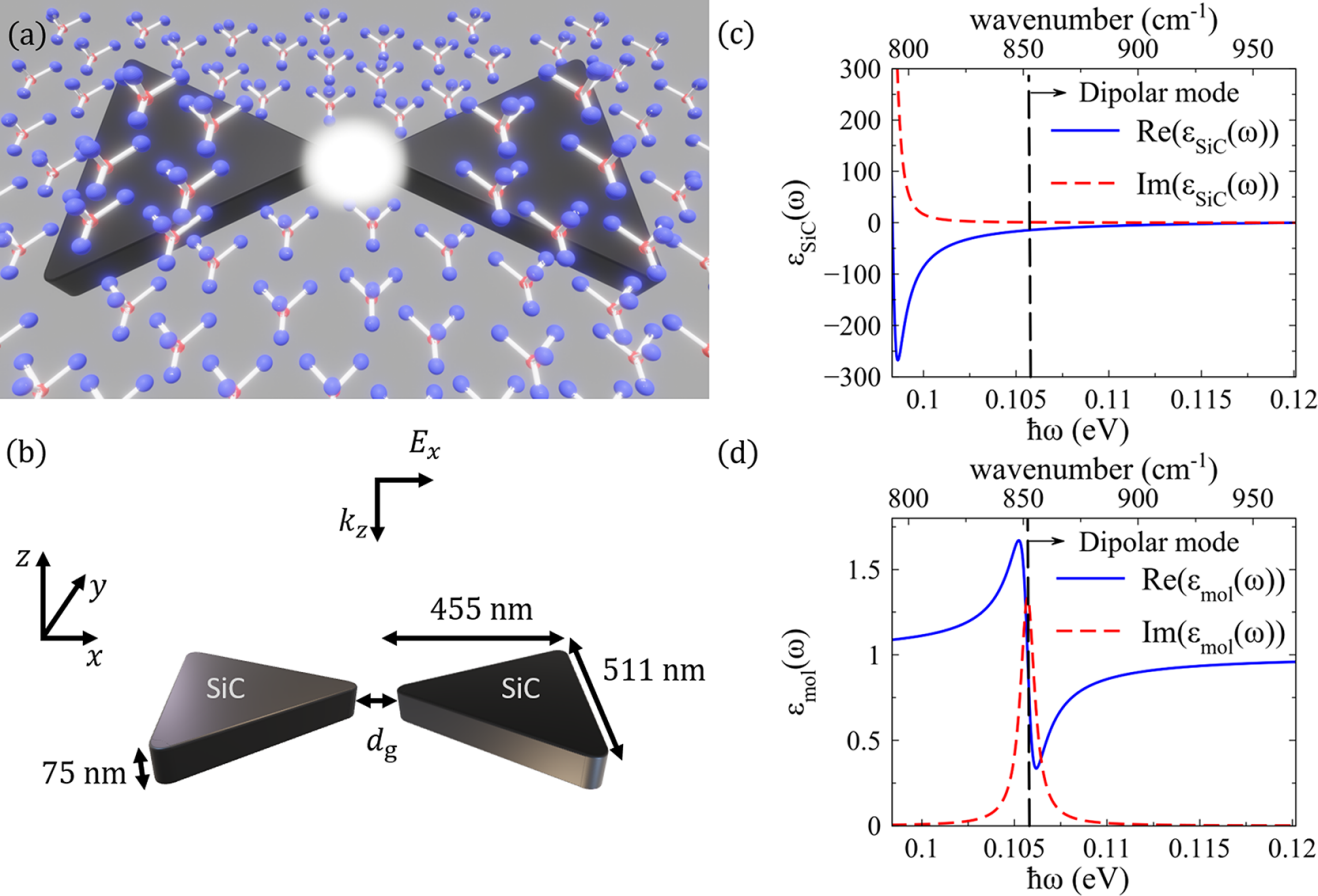
Phononic system under study. (a) Schematic representation of the phononic system, consisting of a SiC bowtie nanoantenna that interacts with nearby molecules. The nanoantenna is composed of two triangular prisms separated by a nanogap. The white region in the sketch represents the strong field enhancement at this nanogap. (b) Geometry and dimensions of the phononic nanoantenna in the absence of molecules. The bowtie nanoantenna is made of SiC and is located in vacuum. The prisms that correspond to each of the two arms of the nanoantenna have the following dimensions: 455 nm long, 511 nm wide, and 75 nm thickness, with a variable nanogap separation *d*_g_ between the two arms ranging from 10 to 100 nm. In each prism of the nanoantenna, the three corners are rounded with a radius of 30 nm, and the edges are rounded with a radius of 15 nm. The bowtie is illuminated by a plane wave polarized along the axis of the bowtie (*x* direction) and propagating along the -*z* direction normal to the nanoantenna (see axis in the sketch). (c) Real (blue solid line) and imaginary (red dashed line) parts of the dielectric function of SiC *ε*_SiC_(ω) (eq 1) plotted as a function of the photon energy (bottom axis) and the wavenumber (upper axis). (d) Real (blue solid line) and imaginary (red dashed line) parts of the dielectric function of the molecules *ε*_mol_(ω) (eq 2, with **ℏ** *ω*_mol_ = 0.106 eV) plotted as a function of the photon energy (bottom axis) and the wavenumber (upper axis). The energy **ℏ** *ω*_mol_ = 0.106 eV is indicated by the vertical black dashed line in panels (c-d) and it is chosen to correspond to the phononic mode of the bare bowtie nanoantenna analyzed in Figure 2a.

The dielectric function of the molecules is also modeled using a Lorentzian function:

2 $$\varepsilon_{\mathrm{mol}}\left(\omega \right)=\varepsilon_{\mathrm{mol},\infty }\left[1+\frac{S^{2}}{\omega_{\mathrm{mol}}^{2}-\omega^{2}-i\omega \gamma_{\mathrm{mol}}}\right]$$

which assumes that the optical response of the molecules is dominated by a single vibrational mode. Here, γ_mol_ is the damping rate of the vibration, *S* is the strength of the oscillator, ω_mol_ the resonant frequency of the vibrational mode, and *ε*_mol,∞_ = 1 the high-frequency permittivity of the molecules. Instead of selecting a specific molecule, the values of *ε*_mol,∞_ = 1, *ℏγ*_mol_ = 0.94 meV, **ℏ** *S* = 0.012 eV have been chosen to facilitate the analysis of the response of the bowtie-molecules system. In particular, we consider a large value of *S* (larger than for typical molecules when normalizing by the resonant frequency^12,22^ ω_mol_), which helps to reach the strong coupling regime.

Further, we vary the vibrational frequency ω_mol_ as a function of the nanogap distance *d*_g_ so that this frequency is always tuned to the resonant frequency ω_ph_ of the dipolar phononic mode of the nanoantenna, ω_mol_ = ω_ph_. For instance, for a nanogap of *d*_g_ = 60 nm, ℏ*ω*_mol_ = 0.106 eV. In Figure 1d we plot the real and the imaginary parts of the dielectric function of the molecules as a function of the photon energy for **ℏ** *ω*_mol_ = 0.106 eV. The imaginary part shows a clear peak at ω_mol_ (frequency indicated by the vertical black dashed line in Figure 1c,d).

### 3.2 Coupled Harmonic Oscillator Model Phononic for Nanoantennas

Before analyzing the full bowtie-molecules system, we perform numerical simulations of the infrared response of the isolated SiC phononic bowtie nanoantenna, i.e., without considering molecules, for a nanogap distance of *d*_g_ = 60 nm. We show in Figure 2a the spectrum of the extinction cross-section σ_ext_ (blue solid line) and of the field enhancement at the center of the nanogap |E_c_/E_0_| (red dashed line, where E_c_ is the amplitude of the total field) under the plane wave illumination. Both spectra are dominated by a narrow lowest-energy peak at ≈0.106 eV (marked by the vertical black dashed line in Figure 2a). For this phononic mode, the resonant field enhancement is |E_c_/E_0_| ≈ 150. The surface charge density (inset in Figure 2a) confirms that this surface phononic mode is dipolar. Other spectral features associated with high-order modes also appear in the extinction and field-enhancement spectra at higher energies, but we focus on the dipolar mode in this work.

**Figure 2:**
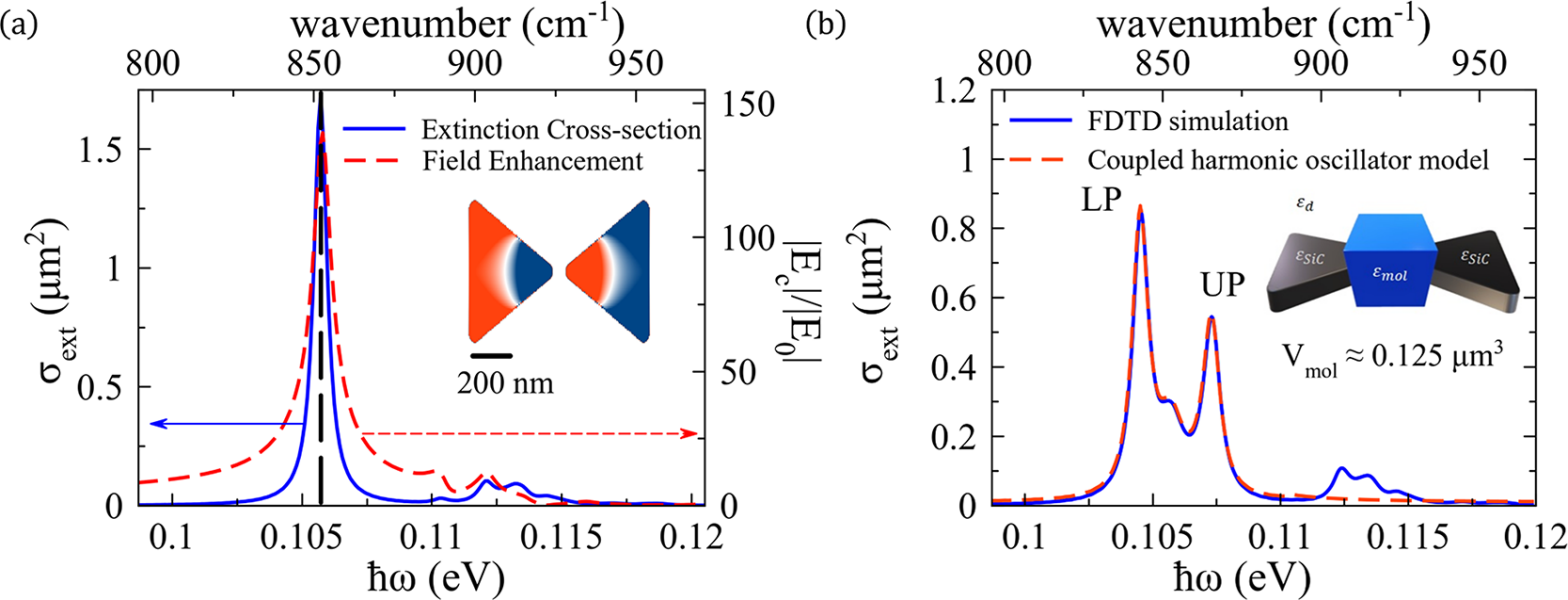
Optical response of a bowtie nanoantenna with nanogap distance *d*_g_ = 60 nm (a) uncoupled and (b) coupled with molecules. (a) Simulated extinction cross-section (blue solid line) and electric field enhancement at the center of the nanogap (red dashed line) of an isolated bowtie nanoantenna illuminated by a plane wave polarized along the bowtie axis (*x* direction in Figure 1b) plotted as a function of the photon energy (bottom axis) and the wavenumber (upper axis). In the figure, the left axis corresponds to the extinction cross-section and the right axis to the electric field enhancement (see arrows). The dipolar phononic mode is found at **ℏ** *ω*_ph_ ≈ 0.106 eV (≈ 855 cm^–1^), marked by the vertical black dashed line. The inset shows the charge density at a given time and at frequency ω_ph_ , where the red and blue (saturated) colors indicate positive and negative charges, respectively. (b) Extinction cross-section of a bowtie nanoantenna coupled with molecules for the same illumination as in (a), plotted as a function of the photon energy (bottom axis) and the wavenumber (upper axis). The blue solid line corresponds to numerical simulations, and the red dashed line to the fit with the coupled harmonic oscillator model (Eqs 3 and 6). The peaks labeled LP and UP correspond to the lower and upper polaritons with energy ≈ 0.1045 eV (≈ 842.8 cm^–1^), and ≈ 0.1073 eV (≈ 865.4 cm^–1^), respectively. The inset shows a scheme of the bowtie nanoantenna coupled to a cubic distribution of molecules occupying a volume V_mol_ (blue cubic region) in vacuum *ε*_d_ = 1. The coupled spectra in (b) have been obtained for V_mol_ ≈ 0.125 μm^3^, which results in a fitted collective coupling strength of **ℏ** *g*_ho_ ≈ 1.39 meV (11.2 cm^–1^).

We study next the coupling of the dipolar mode of this bowtie nanoantenna with the vibrational modes of the surrounding molecules. As discussed in Section 3.1, the molecules are modeled as a homogeneous medium of dielectric function *ε*_mol_(ω) that occupies a cubic region of $V_{mol}\approx L_{mol}^{3}$ centered in the middle of the nanogap (this region is represented as a blue volume in the inset in Figure 2b). To guide the reader about the use of the different volumes involved, Section 2 provides a detailed description of each volume discussed in this work. The extinction cross-section of the nanoantenna coupled to the molecules is shown in Figure 2b for V_mol_ ≈ 0.125 μm^3^ (blue solid line). We observe two well separated peaks, near 0.1045 and 0.1073 eV, which correspond to the lower (LP) and upper (UP) polaritonic excitations, respectively. These polaritons emerge due to the strong coupling^16,60^ (as confirmed below) between a phononic surface mode of the IR nanoantenna and a bright collective vibrational mode involving many molecules.^61–63^ The peak near 0.106 eV between the LP and UP originates from dark collective vibrational modes that do not couple efficiently with the dipolar mode of the nanoantenna.^61^ The resonant frequency of the dark modes is close to that of the uncoupled molecules ω_mol_, which explains why the corresponding peak is near **ℏ** *ω*_mol_ ≈ 0.106 eV. We emphasize that, although we call these modes dark because they do not couple with the dipolar nanoantenna mode, they can still be excited directly by the laser or couple with the tail of high-order nanoantenna modes. We describe the IR response of the system through a coupled harmonic oscillator model.^18,45,47–50^ In this model, we use three equations to reproduce the spectrum. The first two equations are coupled, and describe the response of the bowtie nanoantenna dipolar resonance coupled to the bright collective vibrational mode. The third separate equation describes the response of an uncoupled dark collective vibrational mode. The resulting system of equations is

3 $${ẍ}_{\mathrm{ph}}+\kappa_{\mathrm{ph}}{ẋ}_{\mathrm{ph}}+\omega_{\mathrm{ph}}^{2}x_{\mathrm{ph}}-2g_{\mathrm{ho}}{ẋ}_{m}=F_{\mathrm{ph}}\left(t\right)$$

3b $${ẍ}_{m}+\gamma_{m}{ẋ}_{m}+\omega_{m}^{2}+2g_{\mathrm{ho}}{ẋ}_{\mathrm{ph}}=0$$

$${ẍ}_{m^{\prime }}+\gamma_{m^{\prime }}{ẋ}_{m^{\prime }}+\omega_{m^{\prime }}^{2}=F_{m^{\prime }}\left(t\right)$$ 3c

Equation 3 describes the response of the bowtie nanoantenna, where **x**_ph_ represents the strength of the dipolar phononic mode of the nanoantenna and **x**_m_ that of the bright collective vibrational mode of the molecules. The dots represent the time derivative, and ω_ph_ and κ_ph_ are the frequency and decay rate of the dipolar phononic mode, respectively. **F**_ph_(*t*) corresponds to the effective force driving this mode, which is proportional to the electric field of the external excitation source. Last, *g*_ho_ corresponds the coupling strength between the dipolar phononic mode of the nanoantenna and the bright collective vibrational mode of the molecules (the collective coupling strength). The calculation of the value of this parameter is one of the main purposes for the use of coupled harmonic oscillator model. We note that the coupling term (−2*g*_ho_ ẋ _m_) is proportional to the time derivative of **x**_m_ , which is consistent with related work describing such systems.^45,47^ Alternatively, this model could also be implemented by coupling the positions of the oscillators.^18,48,49^ Indeed, it can be shown that both approaches are almost equivalent under the coupling strength conditions studied here.^50^

Further, eq 4 describes the response of the collective vibrational bright mode of resonant frequency ω_m_ and damping rate γ_m_ , coupled to the IR nanoantenna mode through the term proportional to *g*_ho_. This equation does not include any driving force because we assume that the near fields of the nanoantenna at the position of the molecules are much larger than the external electromagnetic field. The uncoupled dark collective vibrational mode is described using eq 5, where **x**_m′_, γ_m′_, ω_m′_ and **F**_m′_(*t*) represent its strength, decay rate, frequency and effective force driving the oscillator motion (we only consider here a single dark mode for simplicity). As discussed above, these collective vibrational modes need to be considered because, although they do not couple with the dipolar nanoantenna mode, they can be excited directly by the laser or by strongly detuned nanoantenna modes.

We solve eq 3 in the frequency domain by assuming a harmonic time dependence of the form *e* ^–*i*ω*t*^, so that, e.g., **x**_ph_(*t*) = Re­(**x**_ph_ *e* ^–*i*ω*t*^) and **F**_ph_(*t*) = Re­(**F**_ph_ *e* ^–*i*ω*t*^). The extinction cross-section calculated numerically is then obtained from the total dissipated average (⟨ ⟩) power of the oscillators through^22,47^

σext∝⟨Fph(t)·ẋph(t)+Fm′(t)·ẋm′(t)⟩=Im[ω|Fph|2(−ω2−iγmω+ωm2)2[(−ω2−iκphω+ωph2)(−ω2−iγmω+ωm2)−4gho2ω2]+ω|Fm′|22(−ω2−iγm′ω+ωm′2)] 4

where the first term on the right-hand side of the equation originates from the coupling of the bright collective vibrational mode involving many molecules to the dipolar mode of the bowtie, and the second term from the uncoupled dark collective modes. Im­[*x*] indicates the imaginary part of *x*.

We use eq 6 to fit the simulated extinction cross-section spectrum of the coupled system in Figure 2b. For this fit, we fix **ℏ** *κ*_ph_ = 1.1 meV and **ℏ** *ω*_ph_ = 0.106 eV from the simulations of the isolated nanoantenna in Figure 1b, as well as the loss rate of the collective molecular vibrations to **ℏ** *γ*_m_ = **ℏ** *γ*_m′_=**ℏ** *γ*_mol_ = 0.94 meV. We do not consider any shift of ω_ph_ because *ε*_mol,∞_ = 1 in our system. The remaining variables are the fitting parameters (**F**_ph_, **F**_m′_, ω_m_ , ω_m′_ and *g*_ho_), where we consider that ω_m_ and ω_m′_ can be slightly shifted from the value of ω_mol_ used to characterize the dielectric function of the molecules (eq 2) due to the optical coupling with higher-order phononic modes. The comparison of the numerical (blue solid line) and fitted (red dashed line) values in Figure 2b shows that the fitting is very good in the frequency range where the signature of strong coupling emerges (the value of the fitting parameters for all fits in the main text can be found in Section S1 of the Supporting Information). The coupling strength obtained from the fitting in Figure 2b is **ℏ** *g*_ho_ ≈ 1.39 meV, which confirms that the system is in the strong coupling regime when considering the typical criterion of strong coupling,^16,55,60^

5 $$g_{\mathrm{ho}}>\frac{\kappa_{\mathrm{ph}}+\gamma_{\mathrm{mol}}}{4}$$

which is clearly satisfied in our case ( $\frac{\hbar \kappa_{\mathrm{ph}}+\hbar \gamma_{\mathrm{mol}}}{4}$ =0.51 meV).

### 3.3 Semianalytical Treatment of Collective Coupling Strength

In the previous section, we have obtained the coupling strength *g*_ho_ by fitting the extinction cross-section of the bowtie nanoantenna-molecules system using a coupled harmonic oscillator model (eqs 3 and 6). However, this methodology can be computationally expensive when performing a systematic analysis of the coupling with different amounts or spacial distributions of molecules, because a new numerical simulation and fit is required for each case. Further, obtaining a good fit is sometimes challenging. Here, we follow ref 45 (similar approaches can be found in refs 13, 64–66) to obtain a semianalytical expression of the coupling strength based on the microscopic interaction between a phononic mode of a nanoantenna and a vibrational collective mode involving many molecules. This approach allows for systematically obtaining the collective coupling strength by using as input the fields induced by the bare nanoantenna. We emphasize that a single simulation of the bare nanoantenna is enough to obtain the collective coupling strength for any molecular distribution. To ensure this, we assume *ε*_mol,∞_ = 1, which is enough to obtain important insights into the coupling between different types of nanoantennas and molecular distributions, and might be generalized to *ε*_mol,∞_ ≠ 1. In this more general case, to be able to perform a complete analysis with a single simulation, it would be necessary to model the effect of *ε*_mol,∞_ ≠ 1 on the spatial field distribution of the nanoantenna modes.

To obtain the sought expression, we consider a Hamiltonian of N_mol_ molecules, each of them with a vibrational frequency ω_mol_ coupled to a nanoantenna supporting a single phononic mode of frequency ω_ph_. We focus here on the main steps of the derivation, and additional details can be found in Section S2 of the Supporting Information. The Hamiltonian of the coupled system can be written as

6 $$Ĥ=\hbar \omega_{\mathrm{ph}}{â}^{†}â+\overset{N_{\mathrm{mol}}}{\underset{j=1}{\sum }}\hbar \omega_{\mathrm{mol}}{\mathrm{b̂}}_{j}^{†}{\mathrm{b̂}}_{j}+\overset{N_{\mathrm{mol}}}{\underset{j=1}{\sum }}\hbar g_{\mathrm{sa}}^{\left(j\right)}\left({\mathrm{b̂}}_{j}{â}^{†}+{\mathrm{b̂}}_{j}^{†}â\right)$$

where ${â}^{†}$ and â are the creation and annihilation operators of the phononic mode of the nanoantenna, and ${\mathrm{b̂}}_{j}^{†}$ and b̂ _*j*_ are the creation and annihilation operators of the vibrational mode of the molecule *j* (assumed to be harmonic). In this methodology, $g_{\mathrm{sa}}^{\left(j\right)}$ is the real-valued coupling strength^19^ between the phononic mode and the vibration of the molecule *j*. Direct interaction between molecular vibrations are neglected.^67^ $g_{\mathrm{sa}}^{\left(j\right)}$ can be obtained from the product between the transition dipole moment μ of the molecular vibrations and the amplitude of the quantized electric field^68^ Ê ­(**r**_*j*_) acting on the molecule *j*. The former can be connected with the isotropic molecular dielectric function in eq 2, giving:

7 $$g_{\mathrm{sa}}^{\left(j\right)}=\sqrt{\frac{\omega_{\mathrm{ph}}V_{\mu }S^{2}}{4\omega_{\mathrm{mol}}V_{\mathrm{qst}}^{\mathrm{eff}}}}\frac{\left|E^{s}\left(r_{j}\right)\right|}{\left|E_{\max}^{s}\right|}$$

where V_*u*_ is the effective volume that each molecule occupies,|**E** ^*s*^(**r**_*j*_)| is the amplitude of the scattered field at the position of the molecule *j*, $\left|E_{max}^{s}\right|$ corresponds to the maximum amplitude of the scattered electric field, and $V_{\mathrm{qst}}^{\mathrm{eff}}$ is the effective mode volume obtained from the dependence of

Vqst=12∫dVint(ε0(Re(εr(ωph))+2ωphγSiC⁡Im(εr(ωph)))|Es(r)|2+μ0|Hs(r)|2)ε0|Emaxs|2, 8

on the cubic volume of integration V_int_ (see below). In these equations, the amplitudes of the scattered electric and magnetic fields (|**E** ^s^(**r**)| and |**H** ^s^(**r**)|, respectively), do not include the field of the excitation plane wave. The volume of integration V_int_ extends over the interior of the bowtie nanoantenna (dielectric constant *ε*_r_(ω_ph_) = *ε*_SiC_(ω_ph_)) and the surrounding medium (*ε*_*r*_(ω_ph_) = 1). The numerator in eq 10 corresponds to the total electric and magnetic energy of the nanoantenna mode.^69,70^ For phononic materials, as those described by eq 1, the electric energy is considered to be proportional to (Re(εSiC(ωph))+2ωphγSiC⁡Im(εSiC(ωph))) , instead of the often used $\frac{d\left(\omega \varepsilon_{\mathrm{SiC}}\left(\omega \right)\right)}{d\omega }$ evaluated at ω_ph_. Both expressions result in almost identical results, except for resonances near the transverse phononic frequency ω_*t*_, where the latter becomes incorrect.^69–71^

In a rigorous treatment, eq 10 is only applicable to a phononic normal mode^72,73^ characterized by the absence of radiation of photons to the far field. If this condition is not verified, the integral in eq 10 is infinite. This difficulty can be circumvented by using a quasi-normal mode (QNM) treatment^74–76^ but here we follow the method implemented by Koenderink in ref 77 which works very satisfactorily for systems dominated by a single weakly-radiative nanoantenna mode.^44^ This method consists in subtracting the radiative part of eq 10 by a simple linear fit.

To this end, we first evaluate the integral in eq 10 in a cubic domain of increasing volume V_int_, centered in the middle of the nanogap (represented schematically by the blue light dashed line in the inset of Figure 3a). We show in Figure 3a the results of the integration as a function of the length of the side of the integration cubic volume $V_{\mathrm{int}}^{1/3}$ for the same bowtie nanoantenna considered in Section 3.2 (with *d*_g_ = 60 nm). The result of the integral increases rapidly with integration volume V_int_ up to $V_{\mathrm{int}}^{1/3}\approx 1\mu m$ , corresponding to the region of strong near fields inside and near the nanoantenna. For larger integration volumes, a linear increase with a very small slope is observed, due to the contribution of the scattered fields. The effective mode volume $V_{\mathrm{qst}}^{\mathrm{eff}}$ is obtained by subtracting this small linear contribution to the total integral, and taking the limit $V_{\mathrm{int}}^{1/3}\to \infty$ , resulting in a value $V_{\mathrm{qst}}^{\mathrm{eff}}\approx 1.5\times 10^{-3}$ μm^3^ (given graphically by the cut of the red dashed fitted line and the zero value of the *x*-axis in Figure 3a).^77^ We discuss in Section 3.7 an extension of this method that can also be applied to strongly radiative nanoantennas.

**Figure 3:**
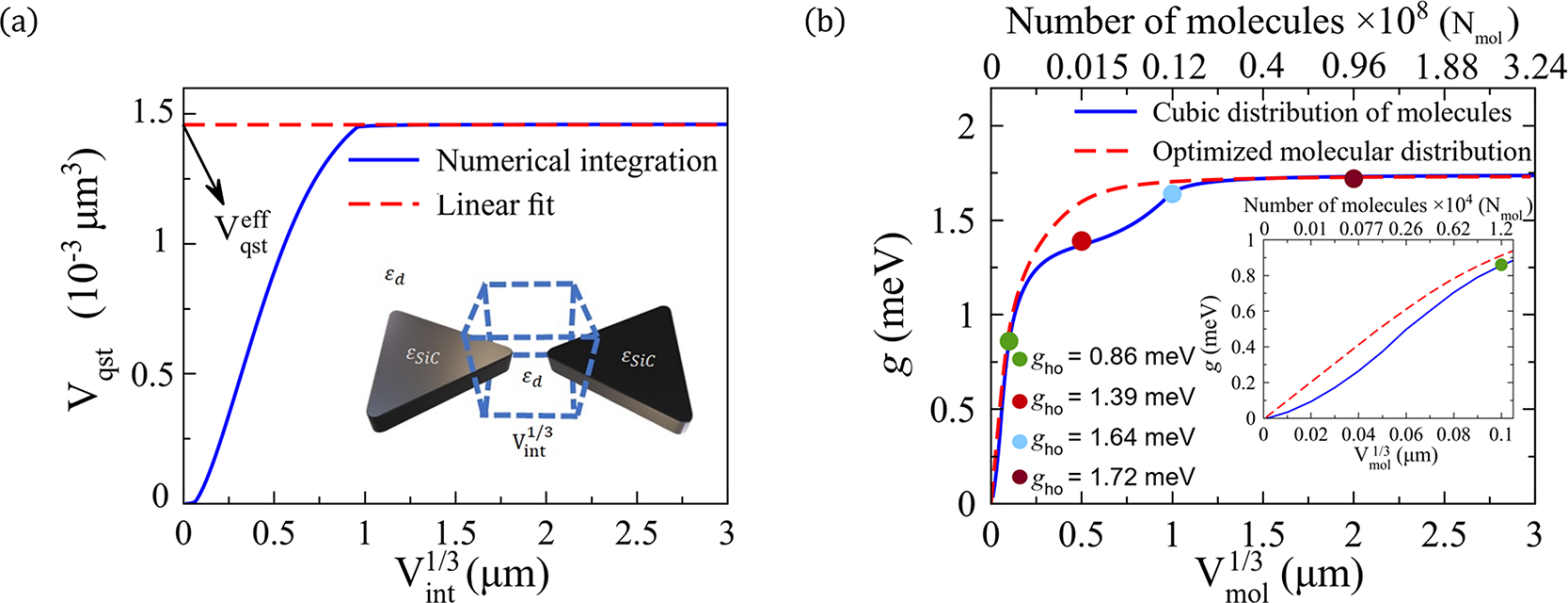
Semianalytical treatment of the coupling between the dipolar phononic mode of the SiC bowtie nanoantenna (nanogap distance *d*_g_ = 60 nm) and the collective vibration of the surrounding molecules. (a) Numerical integration of eq 10, V_qst_, as a function of the length of the cube side $V_{\mathrm{int}}^{1/3}$ of the integration volume, necessary to obtain the effective mode volume $V_{\mathrm{qst}}^{\mathrm{eff}}$ . The linear fitting with $V_{\mathrm{int}}^{1/3}$ for large integration volume is shown by the red dashed line. The effective mode volume is $V_{\mathrm{qst}}^{\mathrm{eff}}\approx 1.5\times 10^{-3}$ μm^3^, which corresponds to the value of this linear fitting at $V_{\mathrm{int}}^{1/3}=0$ (indicated by the black arrow). The inset shows a sketch of the bare bowtie nanoantenna in vacuum and of the cubic integration volume V_int_ (blue light dashed line) used to calculate the integral. (b) Solid and dashed lines: Collective coupling strength *g*_sa_ between the dipolar phononic mode and the bright collective vibrational mode, obtained using eq 14 and plotted as a function of the cubic root of the volume occupied by the molecules $V_{\mathrm{mol}}^{1/3}$ (bottom axis) and of the number of molecules N_mol_ in this volume (upper axis). The blue solid line corresponds to the results for a cubic distribution of molecules, while the red dashed line are obtained for the optimized molecular distribution that maximizes *g*_sa_. The collective coupling strength calculated with both distributions saturates at **ℏ** *g*_sa_ ≈ 1.76 meV (≈14.2 cm^–1^). Color dots: Corresponding values of the coupling strength *g*_ho_ obtained by fitting the extinction cross-section spectrum of the simulated bowtie nanoantenna-molecules system within a coupled harmonic oscillator model following the methodology in Section 3.2. In these simulations, we consider the cubic spatial distribution of the molecules, with green, red, light blue, and maroon dots corresponding to the different volumes V_mol_ ≈ 0.001 μm^3^, V_mol_ ≈ 0.125 μm^3^, V_mol_ ≈ 1 μm^3^, and V_mol_ ≈ 8 μm^3^ respectively. The inset shows a zoom of the collective coupling strength calculated for small volumes (small number of molecules).

To give a more intuitive picture of the volume represented by $V_{\mathrm{qst}}^{\mathrm{eff}}$ , we define a reference volume V_gap_ that is approximately the size of the nanogap. This nanogap region volume is defined as

9 $$V_{\mathrm{gap}}=d_{g}t\sqrt{rd_{g}}=r^{1/2}d_{g}^{3/2}t$$

which is set by the nanoantenna thickness *t* = 0.075 μm, the nanogap distance *d*_g_ and the approximate lateral extension of the hot spot^78,79^ $\sqrt{rd_{g}}$ , with *r* = 0.03 μm the bowtie tip radius. For the value *d*_g_ = 0.06 μm considered here, $V_{\mathrm{gap}}=r^{1/2}d_{g}^{3/2}t\approx 1.91\times 10^{-4}$ μm^3^ and the effective mode volume is approximately $V_{\mathrm{qst}}^{\mathrm{eff}}\approx 8V_{\mathrm{gap}}$ .

To obtain the collective coupling strength of the bowtie nanoantenna-molecules system, the next step consists in expressing the Hamiltonian in eq 8 in the base of the collective vibrational modes involving many molecules.^62,80^ The lowering operator of the bright vibrational collective mode is ${\mathrm{B̂}}_{1}=\overset{N_{\mathrm{mol}}}{\underset{j=1}{\sum }}c_{1j}{\mathrm{b̂}}_{j}=\overset{N_{\mathrm{mol}}}{\underset{j=1}{\sum }}\frac{g_{\mathrm{sa}}^{\left(j\right)}}{\sqrt{\underset{k}{\sum }{|g_{\mathrm{sa}}^{\left(k\right)}|}^{2}}}{\mathrm{b̂}}_{j}$ . There are also N_mol_-1 dark vibrational collective modes described by ${\mathrm{B̂}}_{m\geq 2}=\overset{N_{\mathrm{mol}}}{\underset{j=1}{\sum }}c_{mj}{\mathrm{b̂}}_{j}$ , which do not couple with the dipolar phononic mode and yield the peak at ≈0.106 eV in Figure 2b. Focusing on the bright vibrational mode, the resulting Hamiltonian is

10 $$Ĥ=\hbar \omega_{\mathrm{ph}}{â}^{†}â+\hbar \omega_{\mathrm{mol}}{\mathrm{B̂}}_{1}^{†}{\mathrm{B̂}}_{1}+g_{\mathrm{sa}}\left({\mathrm{B̂}}_{1}{â}^{†}+{\mathrm{B̂}}_{1}^{†}â\right)$$

where *g*_sa_ is the sought collective coupling strength between the dipolar phononic mode and the collective vibrational mode given by

11 $$g_{\mathrm{sa}}=\sqrt{\overset{N_{\mathrm{mol}}}{\underset{j=1}{\sum }}{|g_{\mathrm{sa}}^{\left(j\right)}|}^{2}}$$

Substituting eq 9 into eq 13, we obtain

12 $$g_{\mathrm{sa}}=\sqrt{\frac{\omega_{\mathrm{ph}}S^{2}\int dV_{\mathrm{mol}}{|E^{s}\left(r\right)|}^{2}}{4\omega_{\mathrm{mol}}{|E_{\max}^{s}|}^{2}V_{\mathrm{qst}}^{\mathrm{eff}}}}$$

where we have replaced the sum in eq 13 by an integral after assuming a continuous distribution of molecules. In conclusion, to calculate the collective coupling strength between the dipolar phononic mode and the collective vibrational mode, we calculate the value of the effective mode volume, $V_{\mathrm{qst}}^{\mathrm{eff}}$ and evaluate the integral in the numerator of eq 14 over the volume V_mol_ occupied by the molecules. Additionally, we subtract the slow linear increase with $V_{\mathrm{mol}}^{1/3}$ obtained for $V_{\mathrm{mol}}^{1/3}\to \infty$ to the value of *g*_sa_^2^ obtained by direct integration, following an equivalent procedure as for $V_{\mathrm{qst}}^{\mathrm{eff}}$ .

The resulting coupling strength *g*_sa_ for a cubic volume of molecules of increasing size is shown in Figure 3b (blue solid line) as a function of $V_{\mathrm{mol}}^{1/3}$ (bottom axis, approximately corresponding to the length of the side of the cube) and the number of molecules N_mol_ (upper axis) that occupy this volume. The number of molecules is calculated as N_mol_ = ρ_mol_V_mol_, where we assume that the density of the molecules in solution^81^ is ρ_mol_ = 1.2 × 10^7^ molecules/μm^3^.

The coupling strength increases very fast for small (and increasing) $V_{\mathrm{mol}}^{1/3}$ (see inset in Figure 3b) because a large fraction of the molecules is placed in regions of strong near field (nanogap region). This fast increase occurs until $V_{\mathrm{mol}}^{1/3}\approx 0.15$ μm (N_mol_ ≈ 4.05 × 10^4^ molecules; V_mol_ ≈ 17.67 V_gap_ with V_gap_ the volume of the nanogap region defined in eq 11). *g*_sa_ increases fast again between $V_{\mathrm{mol}}^{1/3}\approx 0.8-1$ μm (N_mol_ ≈ 0.61 × 10^7^–1.2 × 10^7^ molecules; V_mol_ ≈ 2.68 × 10^3^ V_gap_–5.24 × 10^3^ V_gap_) because of the strong fields present in the corners of the nanoantenna of 30 nm radius. For $V_{\mathrm{mol}}^{1/3}$ larger than 1 μm, the coupling strength saturates to **ℏ** *g*_sa_ = 1.76 meV, corresponding to the value obtained when the surrounding space is fully filled with molecules.

To confirm the validity of eq 14, we compare the collective coupling strength *g*_sa_ (blue solid line in Figure 3b) with the values of the coupling strength *g*_ho_ obtained using the procedure described in Section 3.2, where the simulated spectra is fitted using the coupled harmonic oscillator model. The latter are obtained for four different amounts of molecules, corresponding to V_mol_ ≈ 0.001 μm^3^ (green dot), V_mol_ ≈ 0.125 μm^3^ (red dot; configuration analyzed in Figure 2b), V_mol_ ≈ 1 μm^3^ (light blue dot), and V_mol_ ≈ 8 μm^3^ (maroon dot). The fits are shown in Figure 2b and in Figure S1 of the Supporting Information, and additionally, the exact value of the fitting parameters are given in Section S1 of the Supporting Information. Notice that, in this work, we use the generic symbol *g* when comparing *g*_ho_ and *g*_sa_, for example, as in Figure 3b. We find an excellent agreement between the results obtained with these two different methods. We emphasize that eq 14 enables to calculate the coupling strength as a function of number of molecules with a single simulation of the bare nanoantenna.

To further highlight the advantages of using the semianalytical eq 14, we consider next the molecular distribution that will result in the maximum possible collective coupling strength for a given number of molecules. With this purpose, we add molecules in a consecutive manner at the positions where the amplitude of the near electric field of the nanoantenna is most intense (we place the first molecules in the region of strongest fields, the following ones in the region of second strongest fields, and so on until the full occupancy of V_mol_ is reached). The red dashed line in Figure 3b quantifies the value of this optimized coupling strength, which can be compared with the result obtained with the cubic molecular distribution (blue solid line). The former value is approximately 33% larger than the latter for $V_{\mathrm{mol}}^{1/3}=0.05$ μm and 9% larger for $V_{\mathrm{mol}}^{1/3}=0.08$ μm (see inset in Figure 3b). Finally, the collective coupling strength saturates when $V_{\mathrm{mol}}^{1/3}\to \infty$ at the same value *ℏg*_sa_ ≈ 1.76 meV for the two different distributions, as the molecules cover the whole volume outside the nanoantennas in both cases. Additionally, these results indicate that, according to eq 7, the bowtie nanoantenna-molecules system reaches the strong coupling regime for $V_{\mathrm{mol}}^{1/3}≳0.06$ μm (N_mol_ ≳ 2.6 × 10^3^ molecules; V_mol_ ≈ 1.13 V_gap_ with V_gap_ the nanogap region volume) when we consider the cubic distribution of molecules and $V_{\mathrm{mol}}^{1/3}≳0.05$ μm (N_mol_ ≳ 1.5 × 10^3^ molecules; V_mol_ ≈ 0.65 V_gap_) for the optimal distribution of molecules, as better appreciated in the inset in Figure 3b. We thus find that the optimal molecular distribution enhances the collective coupling strength compared to the cubic distribution, but the difference is moderate for the bowtie nanoantenna.

### 3.4 Dependence of the Collective Coupling Strength on the Nanogap Distance

We have considered up to here a fixed nanogap distance of *d*_g_ = 60 nm. We explore in Figure 4 the effects of changing this value on the collective coupling strength *g*_sa_. *g*_sa_ is obtained from the semianalytical eq 14 for an increasing volume occupied by the molecules V_mol_ (or equivalently, increasing number of molecules N_mol_). In this analysis, we consider the optimized molecular distribution that maximizes *g*_sa_. We show in Figure 4a that, for relatively few molecules, *g*_sa_ is appreciably larger when the separation distance *d*_g_ is smaller because small nanogaps localize and enhance electromagnetic fields more efficiently. For example, in the case of $V_{\mathrm{mol}}^{1/3}=0.05$ μm, *g*_sa_ is twice larger for *d*_*g*_ = 10 nm than for *d*_g_ = 100 nm (see the inset in Figure 4a). However, this tendency is reversed for $V_{\mathrm{mol}}^{1/3}≳0.2$ μm, so that, *g*_sa_ becomes weaker for smaller nanogap distances than for the larger ones. The difference in this case is, however, small, so that the collective coupling strength depends weakly on the size of the nanogap when the system couples with many molecules. For instance, for a number of molecules N_mol_ ≳ 1.2 × 10^7^, *g*_sa_ is ≈1.1 times larger for *d*_g_ = 100 nm than for *d*_g_ = 10 nm.

**Figure 4:**
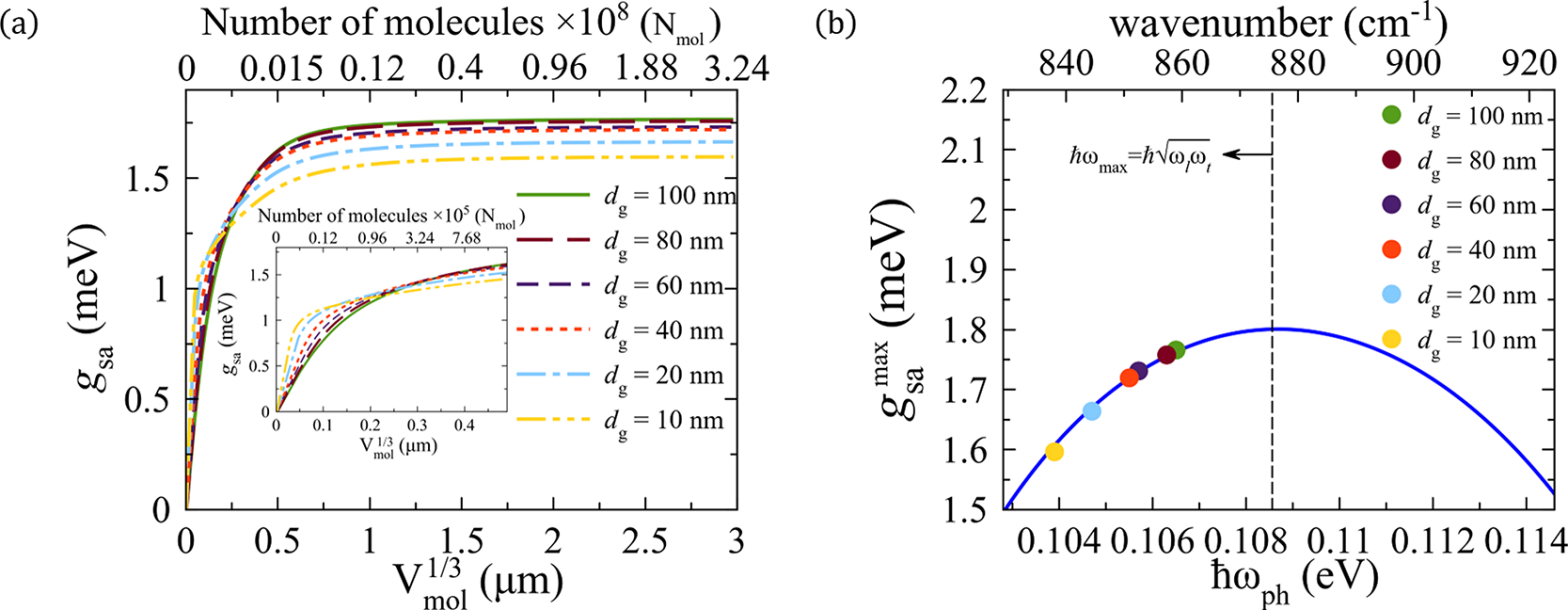
Dependence of the collective coupling strength between the dipolar mode of SiC bowtie nanoantennas and the vibrations of the surrounding molecules on nanogap distances *d*_g_. (a) Collective coupling strength obtained from the numerical integration of eq 14 using the optimized molecular distribution that maximizes *g*_sa_ for a given number of molecules N_mol_, plotted as a function of the cubic root of the volume occupied by the molecules $V_{\mathrm{mol}}^{1/3}$ (bottom axis) and the number of the molecules in this volume N_mol_ (upper axis). These results are obtained for nanogap distances from *d*_g_ = 10 nm to *d*_g_ = 100 nm, as indicated in the legend. The inset shows a zoom of the collective coupling strength calculated for small $V_{\mathrm{mol}}^{1/3}$ . (b) Maximum collective coupling strength plotted as a function of the resonant energy (bottom axis) and wavenumber (upper axis) of the dipolar mode of the phononic nanoantenna. These results are obtained using eq 18 (blue solid line) and from the $V_{\mathrm{mol}}^{1/3}\to \infty$ limit of the numerical results in panel (a) (dots). The maximum of eq 18 is 1.82 meV, reached at $\hbar \omega_{\max}=\hbar \sqrt{\omega_{l}\omega_{t}}\approx 0.1086$ eV. This value is marked by the vertical black dashed line.

To understand the difference for large N_mol_ , we derive next a quasistatic expression of *g*_sa_ valid when the bowtie nanoantenna is fully surrounded by molecules. The quasistatic approximation is justified by the small size of the nanoantenna (compared to the vacuum wavelength).^40,82^ Inserting first eq 10 into eq 14 we obtain

13 gsa=ωphS22ωmol1∫dVmolε0|Es(r)|2+∫dVSiCε0(Re(εSiC)+2ωphγSiC⁡Im(εSiC))|Es(r)|2+∫dVmolμ0|Hs(r)|2+∫dVSiCμ0|Hs(r)|2∫dVmolε0|Es(r)|2

and considering that the contribution of the magnetic field in the denominator of eq 15 is negligible in small phononic and IR plasmonic nanoantennas, the collective coupling strength is then:

14 gsa=ωphS22ωmol1∫dVmolε0|Es(r)|2+∫dVSiCε0(Re(εSiC)+2ωphγSiC⁡Im(εSiC))|Es(r)|2∫dVmolε0|Es(r)|2

We have separated the volume integral of these equations into the contribution from the region outside (V_mol_) and inside the phononic nanoantenna (V_SiC_). Furthermore, we have taken into account that integrating over the region V_mol_ occupied by the molecules is the same, in this case, as integrating over all the volume outside the nanoantenna. Following ref 83 the energy inside and outside the nanoantenna can be connected under the quasistatic approximation using

15 ∫dVmolε0|Es(r)|2=−∫dVSiCε0Re(εSiC)|Es(r)|2

Inserting eq 17 into eq 16, and using eq 1, we finally obtain

16 $$g_{\mathrm{sa}}^{\max}=\sqrt{-\frac{\omega_{\mathrm{ph}}S^{2}}{4\omega_{\mathrm{mol}}}\frac{\left(\omega_{t}^{2}-\omega_{\mathrm{ph}}^{2}\right)\left(\omega_{l}^{2}-\omega_{\mathrm{ph}}^{2}\right)}{\left(\omega_{l}^{2}-\omega_{t}^{2}\right)\omega_{\mathrm{ph}}^{2}}}$$

where we have considered that γ_SiC_≪ ω_ph_ (for further details, see Section S3 of the Supporting Information). The quasistatic eq 18 corresponds to the collective coupling strength between a quasistatic phononic mode and the bright collective vibrational mode of molecules filling all the surrounding space. Remarkably, this coupling strength does not depend directly on the field distribution of the nanoantenna, but only on the dielectric function of SiC and the molecules, and on the resonant frequency of the phononic mode (and thus on the nanogap distance *d*_g_). Thus, we would obtain the same *g*_sa_^max^ for small nanoantennas with different geometries but with the same resonant frequency. In resonance (ω_ph_ = ω_mol_), the maximum of Eq 18 is $\frac{\hbar S}{2}\sqrt{\frac{\omega_{l}-\omega_{t}}{\omega_{l}+\omega_{t}}}=1.82$ meV, which is reached at the dipolar mode energy of $\hbar \omega_{\mathrm{ph}}=\hbar \sqrt{\omega_{l}\omega_{t}}\approx 0.1086$ eV. This is the maximum attainable coupling strength for any small resonant SiC nanoantenna.

In Figure 4b, we show the collective coupling strength calculated using eq 18 (blue solid line) as a function of the phononic mode energy. The dots in the figure indicate the corresponding values of *g*_sa_^max^ obtained from the numerical calculations of the isolated nanoantennas (applying Eq 14) when we consider that the space surrounding the nanoantenna is fully filled with molecules, and the nanogap distance varies from *d*_g_ = 10 nm until *d*_g_ = 100 nm. As expected, the agreement between the two sets of results is very good, confirming that the maximum quasistatic coupling strength is determined by the molecular and phononic dielectric function and by the resonant frequency according to eq 18, and thus it does not directly depend on field confinement.

Last, it is interesting to compare this latter result with that obtained in ref 45, which analyzes the coupling between vibrations and optical modes in dielectric resonators (see also refs 65 and 66). This previous work found that the maximum coupling strength was given exclusively by the properties of the vibrational (or electronic) excitations that couple with the mode of the dielectric resonator, $\hbar g_{\mathrm{sa}}^{\max}=\hbar$ *S*/2, corresponding to 6 meV in our system, and thus it is independent of the properties of the nanoresonator itself. The difference in the coupling with dielectric resonators and with phononic nanoantennas largely occurs because, in the latter situation, a large part of the electric energy is inside the nanoantenna. The fields inside the nanoantenna do not couple with the vibrational excitations, which diminishes the maximum coupling strength by a factor that depends weakly on the resonant frequency. We discuss this point further in Section 3.8, as well as the role played by the magnetic field, when analyzing the coupling with plasmonic modes.

### 3.5 Coupling with Infrared Gold Plasmonic Nanoantennas

In Sections 3.1–3.4, we have considered that molecular vibrations couple with phononic modes of SiC nanoantennas. In this and the following sections, we analyze the coupling between molecular vibrations and IR plasmonic resonances in a gold (Au) bowtie nanoantenna. The nanoantenna is again formed by two prisms with a triangular shape and *t* = 75 nm thickness, illuminated by a plane-wave polarized along the bowtie axis (*x*-axis) and propagating along the −*z* direction, as shown in Figure 5a. However, the lateral size of the triangular prisms are much larger than for the SiC structure (3.78 μm long and 4.20 μm wide) so that the dipolar resonance emerges at similar infrared wavelengths, facilitating the comparison between plasmonic and phononic nanoantennas. The corners of the nanoantenna are rounded with a radius of *r* = 300 nm, and *d*_g_ = 60 nm is again the nanogap distance between the two prisms. Results for *r* = 30 nm are shown in Section S7 of the Supporting Information. We are interested in the response at infrared frequencies, and thus describe the dielectric function of Au using a Drude model:^84,85^

17 $$\varepsilon_{\mathrm{Au}}\left(\omega \right)=\varepsilon_{\mathrm{Au},\infty }-\frac{\omega_{p,\mathrm{Au}}^{2}}{\left(\omega^{2}+i\omega \gamma_{\mathrm{Au}}\right)}$$

where **ℏ** *ω*_p,Au_ = 8.9 eV is the plasma energy, **ℏ** *γ*_Au_ = 75.9 meV is the damping and *ε*_Au,∞_ = 8.5 is the dielectric constant at high frequency.^86^ The dielectric function of the molecules is modeled similarly to the case of phononic nanoantennas, i.e., using eq 2 with **ℏ** *S* = 0.012 eV, **ℏ** *γ*_mol_ = 0.94 meV and *ε*_mol,∞_ = 1. The vibrational frequency ω_mol_ is again chosen so that the system is in resonant condition **ℏ** *ω*_mol_ = 0.106 eV.

**Figure 5:**
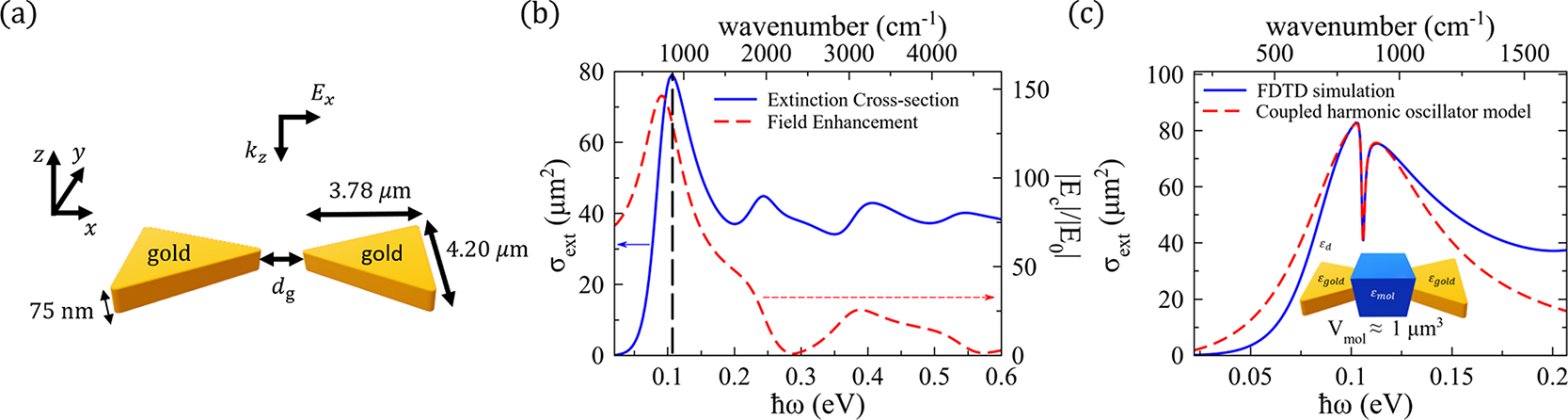
Optical response of a Au plasmonic nanoantenna. (a) Sketch of the gold bare bowtie nanoantenna in vacuum. The two prisms that form the nanoantenna are 3.78 μm long, 4.20 μm width and 75 nm thick with nanogap distance between them *d*_g_ = 60 nm. Each corner is rounded with a radius of 300 nm, and the edges are rounded with a radius of 15 nm. The dielectric function of gold is modeled as a Drude metal according to eq 19, where the parameters are chosen to fit the data in ref 86. The gold bowtie is illuminated by a plane wave polarized along the bowtie axis (*x* direction) and propagating along the -*z* direction normal to the nanoantenna (see axis in the sketch). (b) Infrared response of the bare bowtie nanoantenna in (a) calculated numerically. The extinction cross-section (blue solid line) and the field enhancement (red dashed line) are plotted as a function of the photon energy (bottom axis) and the wavenumber (upper axis). The left axis corresponds to the extinction cross-section and the right axis to the electric field enhancement (see arrows). The vertical black dashed line indicates the energy of the dipolar plasmonic mode **ℏ** *ω*_pl_ ≈ 0.106 eV (≈855 cm^–1^). (c) Infrared response of the gold bowtie nanoantenna in (a) coupled with a cubic distribution of molecules in the nanoantenna gap occupying a volume V_mol_ ≈ 1 μm^3^. The blue line corresponds to the simulation results plotted as a function of the photon energy (bottom axis) and the wavenumber (upper axis). The red dashed line corresponds to the fit obtained from the coupled harmonic oscillator model (eq 6). The molecules are resonant at **ℏ** *ω*_mol_ ≈ 0.106 meV (≈ 855 cm^–1^) and the dip near this energy corresponds to a Fano resonance. The inset shows a sketch of the cubic region occupied by the molecules (blue region). The value of the coupling strength obtained from the fitting is **ℏ** *g*_ho_ ∼ 4.00 meV (∼ 32.26 cm^–1^).

### 3.6 Coupled Harmonic Oscillator Model for Plasmonic Nanoantennas

We show in Figure 5b the extinction cross-section σ_ext_ (blue solid line) and the field enhancement at the center of the nanogap |E_c_/E_0_| (red dashed line) of the bare Au bowtie nanoantenna with *d*_g_ = 60 nm, plotted as a function of the photon energy (bottom axis) and the wavenumber (upper axis). Several plasmonic resonances can be observed in the figure. We focus on the dipolar plasmonic mode with resonant frequency ω_ph_ near 0.106 eV, marked by the vertical black dashed line. The dipolar nature of this lowest-energy mode is confirmed by the surface charge distribution (not shown). The field enhancement at the center of the nanogap is approximately 150 for this mode. The field enhancement and the resonant frequencies are thus similar as for the SiC nanoantenna. On the other hand, the extinction cross-section is 2 orders of magnitude larger because of the much larger dimensions of the Au nanoantenna. Further, the line width of the plasmonic dipolar resonance is also broader, due to the large losses of the plasmonic mode. Some of these losses are connected with ohmic dissipative processes in the metal but, importantly, the large size of the Au nanoantenna also leads to very strong radiative losses.

We consider next the coupling of the dipolar plasmonic mode with the vibration of molecules occupying a cubic volume V_mol_ ≈ 1 μm^3^ centered at the middle of the nanogap (blue cubic region in the inset in Figure 5c). The extinction cross-section in Figure 5c (blue solid line) exhibits a dip in the dipolar plasmonic peak at the resonant frequency of the molecules ω_mol_. The relatively narrow line shape of this dip originates from a Fano-like resonance,^87–90^ which is a fingerprint of the weak coupling regime.^19,91^ To confirm that the system is in this regime, we extract the value of the coupling strength by fitting the numerical results with the expression of the cross-section (eq 6) using the coupled harmonic oscillator model, according to the procedure described in Section 3.2. We fix the value of the parameters defining the plasmonic resonance to those obtained from the simulation of the bare nanoantenna (**ℏ** *κ*_pl_ = 98.51 meV, **ℏ** *ω*_pl_ = 0.106 eV), and the vibrational losses to those of the individual molecules, **ℏ** *γ*_m_ = **ℏ** *γ*_m′_ = **ℏ** *γ*_mol_ = 0.94 meV. The other variables (**F**_pl_ , **F**_m′_, ω_m_ , ω_m′_ and *g*_ho_) are again the fitting parameters, where ω_m_ and ω_m′_ can be slightly shifted from ω_mol_ because of the coupling with high-order plasmonic modes. The fitted cross-section is shown by the red dashed line in Figure 5c. The fit is not good over the whole spectral range due to the contribution of high-order plasmonic modes not included in the coupled harmonic oscillator model, as well as to the influence of large radiative plasmonic losses (which leads to non-Lorentzian modes).^92,93^ However, the fit is good in the critical region near the dip, allowing us to obtain a coupling strength of **ℏ** *g*_ho_ ∼ 4.00 meV (a small correction of this value is discussed in Section 3.7). In this situation, thus, $\frac{g_{\mathrm{ho}}}{\kappa_{\mathrm{pl}}+\gamma_{\mathrm{mol}}}\approx 0.042<\frac{1}{4}$ , which confirms that the system is indeed in the weak coupling regime (eq 7).

### 3.7 Extension of the Semianalytical Treatment to Strongly Radiative Nanoantennas

The objective of this section is to use a similar methodology as that employed for phononic nanoantennas to obtain the coupling strength between noble-metal plasmonic nanoantennas and molecules with an analytical expression that requires a single simulation to analyze a variety of molecular spatial distributions. We first show that the approach developed in Section 3.3 shows significant shortcomings when applied to systems with large radiative losses, and then describe an improved approach.

We show in Figure 6a the integrated volume V_qst_ obtained from eq 10 (blue solid line) as a function of the length of the side of the cubic integration volume $V_{\mathrm{int}}^{1/3}$ for the dipolar mode of the gold bowtie nanoantenna with nanogap distance of *d*_g_ = 60 nm. The integrated volume shows a fast linear increase with $V_{\mathrm{int}}^{1/3}$ for $V_{\mathrm{int}}^{1/3}≳10$ μm, due to the strong radiation of the IR nanoantenna to the far field. When we use a linear fit (red dashed line) to obtain the effective mode volume following ref 77 and Section 3.3, we obtain $V_{\mathrm{qst}}^{\mathrm{eff}}\approx 4\times 10^{-3}$ μm^3^ (corresponding to the value of the linear fit at $V_{\mathrm{int}}^{1/3}=0$ ), which we show below not to be accurate.

**Figure 6:**
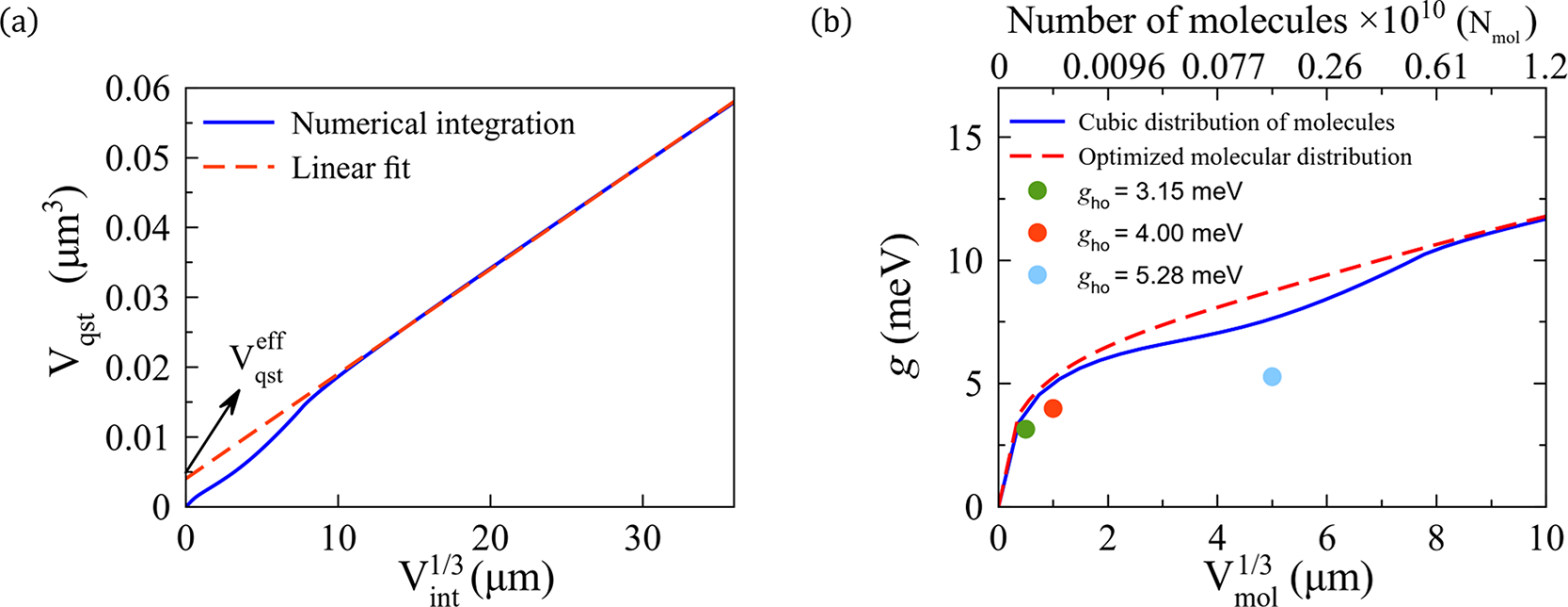
Analysis of the IR gold plasmonic nanoantenna-molecules coupling following the approach validated for weakly radiative quasistatic nanoantennas. We consider the coupling between the dipolar mode of a gold bowtie nanoantenna with nanogap distance of *d*_g_ = 60 nm and the collective vibration of the surrounding molecules. (a) Numerical integration of eq 10 (blue solid line) as a function of the length of the side of the cubic integration volume, $V_{\mathrm{int}}^{1/3}$ , which is used to obtain the effective mode volume $V_{\mathrm{qst}}^{\mathrm{eff}}$ . A linear fit for large integration volume is shown by the red dashed line. The effective mode volume is $V_{\mathrm{qst}}^{\mathrm{eff}}\approx 4\times 10^{-3}$ μm^3^, which corresponds to the value of the linear fit at $V_{\mathrm{int}}^{1/3}$ = 0 (indicated by the black arrow). (b) Collective coupling strength between the dipolar IR plasmonic mode and the bright collective vibrational mode, as a function of the cubic root of the volume occupied by the molecules, $V_{\mathrm{mol}}^{1/3}$ (bottom axis), and the number of molecules N_mol_ in this volume (upper axis). The blue solid line corresponds to the results obtained for a cubic distribution of molecules, while the red dashed line is obtained for the optimized molecular distribution that maximizes *g*_sa_. In both cases, eq 14 is used and *g*_sa_ tends to infinite for large $V_{\mathrm{mol}}^{1/3}$ . The colored dots correspond to the values obtained by fitting the extinction cross-section spectra of the simulated bowtie nanoantenna-molecules system with a coupled harmonic oscillator model, following the methodology in Sections 3.2 and 3.6. The green, red, and light blue dots correspond to cubic molecular distributions of volume V_mol_ ≈ 0.125 μm^3^, V_mol_≈ 1 μm^3^, and V_mol_ ≈ 125 μm^3^, respectively.

We plot next in Figure 6b the collective coupling strength *g*_sa_, as obtained by integrating directly eq 14 following the procedure in Section 3.3, with ω_ph_ = ω_pl_ and γ_SiC_ = γ_Au_. We consider a cubic distribution of molecules of increasing volume V_mol_ (blue line) and the optimized molecular distribution of the same volume (red dashed line). The collective coupling strength obtained in both cases tends to infinite with increasing $V_{\mathrm{mol}}^{1/3}$ , which already highlights the deficiencies of this simple approach. Further, the dots in the figure show the coupling strength *g*_ho_ obtained from a fit to the analytical expression of the cross-section of the nanoantenna-molecules system (eq 6) derived from the coupled harmonic oscillator model (Sections 3.2 and 3.6). These values of *g*_ho_ are obtained for the cubic molecular distribution of volume V_mol_ ≈ 0.125 μm^3^ (green dot), $V_{\mathrm{mol}}\approx 1$ μm^3^ (red dot), and V_mol_ ≈ 125 μm^3^ (light blue dot). The agreement between these two sets of results (obtained from the fit and the analytical equation) is reasonable, but a significant difference still remains. We discuss below a small correction to the value *g*_ho_, but we show in Section S6 of the Supporting Information that this correction does not eliminate the difference between the results obtained from both approaches.

We consider next how to overcome these difficulties by using an improved procedure to better evaluate the effective mode volume and the collective coupling strength for highly radiative nanoantennas. To this aim, we decompose the total dipole induced in the whole nanoantenna into a collection of local dipoles covering different subregions of the nanoantenna. These dipoles are treated as point-like sources, and their far-field radiation is defined as the component of the emitted light that scales with distance *r*_dip_ as 1/*r*_dip_, according to the classical Green’s function.^39,94^ This far-field radiation is then subtracted from the scattered fields (i.e., from the total fields after subtracting the fields of the incident plane wave). The procedure is discussed in more detail in Section S5 of the Supporting Information.

We show in Figure 7a a map of the spatial distribution of the electric field amplitude scattered by the dipolar mode of the bare IR nanoantenna (i.e., in the absence of molecules) in the vertical *y* = 0 plane (see axis in Figure 5a, with *x* = *y* = *z* = 0 the center of the nanogap), normalized by the incident field amplitude. In this panel, the radiative fields have not been subtracted. The *x*-component of the field amplitude (parallel to the bowtie nanoantenna axis) is plotted in this figure because it presents the strongest values. The scattered fields remain significant even far from the nanoantenna. The corresponding nonradiative field obtained after subtracting the far-field radiation is plotted in Figure 7b. The amplitude of this electric field is much smaller far away from the nanoantenna. We apply the same procedure to the magnetic field and calculate the effective mode volume ${Ṽ}_{\mathrm{rdc}}^{\mathrm{eff}}$ through an extension of eq 10 that has been developed to treat the quasi-normal modes (QNMs), characteristic of highly radiative systems.^74–76,95,96^ According to this approach, the effective mode volume is defined as a complex quantity given by^95–98^

18 Ṽrdceff=12∫dVint(ε0Es(r)(Re(εr(ωpl))+2ωplγAu⁡Im(εr(ωpl)))Es(r)−Hs(r)μ0Hs(r))ε0(Emaxs)2,

where the integral in the numerator extends over all volume (*ε*_*r*_(ω_pl_) = 1 outside the nanoantenna and *ε*_Au_(ω_pl_) given by eq 19 inside). Here, the fields **E** ^s^(**r**), and **H** ^s^(**r**) correspond to the scattered fields of the bare nanoantenna after subtracting the far field radiation. The factor (Re(εr(ωpl))+2ωplγAuIm(εr(ωpl))) originates from the expression of the energy density inside the gold at the IR frequencies considered here, which is discussed in Section S4 of the Supporting Information. To illustrate the behavior of the integral, the blue solid line in Figure 7c shows the absolute value of the result of eq 20, performing the integration in this case over a cubic region (centered in the middle of the nanogap) of increasing volume $V_{\mathrm{int}}^{1/3}$ . We denote the result of this integral over a finite volume as Ṽ _rdc_. | Ṽ _rdc_| increases rapidly with $V_{\mathrm{int}}^{1/3}$ from $V_{\mathrm{int}}^{1/3}=0$ μm until $V_{\mathrm{int}}^{1/3}=10$ μm, due to the strong fields near the nanoantenna. The value of | Ṽ _rdc_| obtained for $V_{\mathrm{int}}^{1/3}\to \infty$ corresponds to the absolute value of the effective mode volume $|{Ṽ}_{\mathrm{rdc}}^{\mathrm{eff}}$ | = 6.4 × 10^–3^ μm^3^ ( ${Ṽ}_{\mathrm{rdc}}^{\mathrm{eff}}$ = (5.6 + 3.0i) × 10^–3^ μm^3^). Further, we compare again this value with the volume of the nanogap region V_gap_ defined by eq 11. We obtain V_gap_ = 6.03 × 10^–4^ μm^3^ for this gold plasmonic nanoantenna, so that $\left|{Ṽ}_{\mathrm{rdc}}^{\mathrm{eff}}\right|$ is approximately 10 times larger than V_gap_.

**Figure 7:**
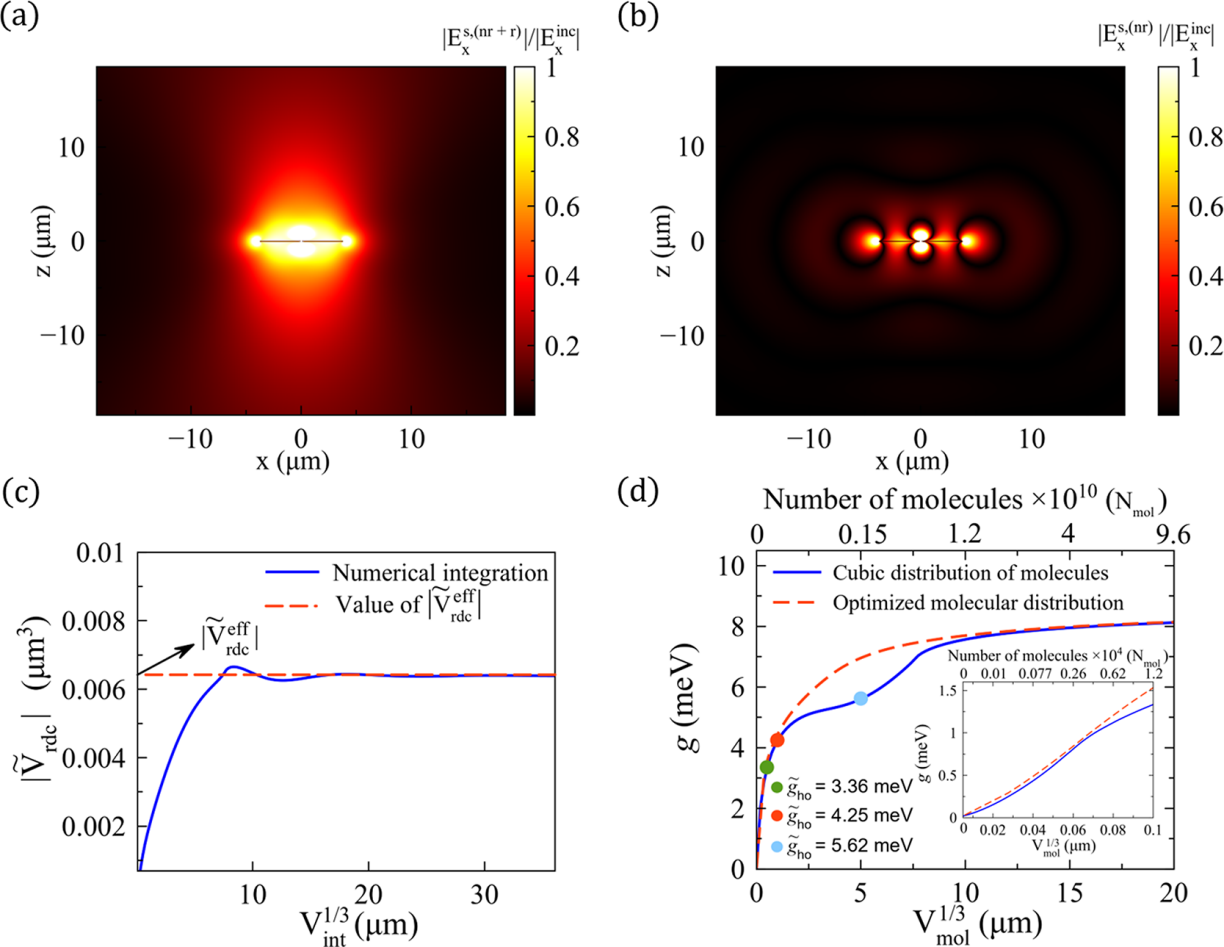
Analysis of the improved determination of the collective coupling strength between molecular vibrations and the dipolar mode of a highly radiative IR gold plasmonic nanoantenna. (a) Spatial distribution in the vertical *y* = 0 plane of the *x*-component of the scattered electric field amplitude (including radiative and nonradiative contributions, but not the excitation) normalized by the amplitude of the incident field (see coordinate axis in Figure 5a; with the nanogap center at *x* = *y* = *z* = 0). The colors are saturated for values larger than 1. (b) Same as (a), after subtracting the radiative fields to obtain the nonradiative contribution, according to the procedure discussed in the text. (c) Absolute value of the numerical integration of eq 20 (blue solid line, |Ṽ_rdc_|), as a function of the length of the side of the integration volume, $V_{\mathrm{int}}^{1/3}$ , for a gold bowtie nanoantenna with nanogap distance of *d*_*g*_ = 60 nm. The red dashed line corresponding to the value of $V_{\mathrm{int}}^{1/3}\to \infty$ , indicates the effective mode volume |Ṽ_rdc_^eff^| = 6.4 × 10^−3^ μm^3^ (Ṽ_rdc_^eff^ = (5.6 + 3.0*i*) × 10^–3^ μm^3^) (see black arrow). (d) Blue solid and red dashed line: collective coupling strength obtained from the numerical integration of eq 14 (after replacing the value of the real mode volume by the absolute value of the complex mode volume as indicated in the text) for a cubic distribution of molecules (blue solid line) and the optimized molecular distribution (red dashed line), as a function of $V_{\mathrm{mol}}^{1/3}$ (bottom axis) and the number of molecules N_mol_ that occupy this volume (upper axis). Dots: values of the collective coupling strength obtained by fitting the extinction cross-section of the molecule-nanoantenna system with eq 6 (derived from the harmonic oscillation model) and using the correction in eq 20 from QNM theory for a complex mode volume. The green, red, and light blue dots correspond to cubic regions of volume V_mol_ ≈ 0.125 μm^3^, V_mol_ ≈ 1 μm^3^, and V_mol_≈125 μm^3^, respectively. The inset shows a zoom to the region of small volumes.

We calculate next the value of the collective coupling strength from eq 14 replacing the value of $V_{\mathrm{qst}}^{\mathrm{eff}}$ by $\left|{Ṽ}_{\mathrm{rdc}}^{\mathrm{eff}}\right|$ . In this case, the electric field in the numerator of eq 14 also corresponds to that obtained after subtracting the far-field radiation. We show in Figure 7d the resulting collective coupling strength *g*_sa_, as calculated using the cubic distribution of molecules V_mol_ (blue solid line). Similarly as in the phononic nanoantenna case, *g*_sa_ grows fast with $V_{\mathrm{mol}}^{1/3}$ (bottom axis) and, equivalently, so does it with the estimated number of molecules N_mol_ (top axis). This fast increase is due to the highly concentrated electromagnetic fields near the corners of the nanoantenna (increase at $V_{\mathrm{mol}}^{1/3}\approx 7.0$ μm, corresponding to N_mol_ ≈ 4.12 × 10^9^ molecules and V_mol_ ≈ 5.69 × 10^5^V_gap_) and, particularly, near the nanogap (for small V_mol_). For $V_{\mathrm{mol}}^{1/3}≳15$ μm, the collective coupling strength almost saturates to the value of **ℏ** *g*_sa_ ≈ 8.4 meV (corresponding to the value where the surrounding space is filled with molecules). This maximum value of the collective coupling strength confirms that this system cannot reach the strong coupling regime because $\frac{g_{\mathrm{sa}}}{\kappa_{\mathrm{pl}}+\gamma_{\mathrm{mol}}}\approx 0.084<\frac{1}{4}$ (eq 7). Importantly, one can observe in the figure that the results calculated with the semianalytical eq 14 are in good agreement with the coupling strength *g̃*_ho_ obtained using the coupled harmonic oscillator model (colored dots, Sections 3.2 and 3.6). We note that, for this comparison, we have corrected the values of *g*_ho_ from the coupled harmonic oscillator model by applying^76,98^

g~ho2=|gho2(1−iIm(Ṽrdceff)Re(Ṽrdceff))| 19

which takes into account that the plasmonic mode is a quasi-normal mode. Here, g̃_ho_ is calculated for volumes V_mol_ ≈ 0.125 μm^3^ (green dot), V_mol_ ≈ 1 μm^3^ (maroon dot), and V_mol_ ≈ 125 μm^3^ (light blue dot), respectively.

Additionally, we plot in Figure 7d the collective coupling strength *g*_sa_ obtained with the semianalytical expressions (including the correction for the strong radiation) when considering the optimized molecular distribution V_mol_ that maximizes this value for a fixed number of molecules (red dashed line). A zoom of the results for small volumes is shown in the inset. The difference between the cubic and optimized molecular distribution is larger for $V_{\mathrm{mol}}^{1/3}\approx 2.5-7.5$ μm (V_mol_ ≈ 2.59 × 10^4^ V_gap_ – 6.99 × 10^5^ V_gap_).

### 3.8 Comparison of Phononic and Plasmonic Infrared Nanoantennas

We analyze next the differences in the coupling of molecules with phononic and IR (noble-metal) plasmonic bowtie nanoantennas. Figure 8a shows the collective coupling strength *g*_sa_ obtained for both types of nanoantennas with *d*_g_ = 60 nm (blue solid line for the gold plasmonic nanoantenna and red dashed line for the phononic nanoantenna), as a function of the size of the cubic volume occupied by the molecules ( $V_{\mathrm{mol}}^{1/3}$ , bottom axis) and the number of molecules (N_mol_, top axis). These results correspond to those plotted in Figure 3b (blue solid line) and Figure 7d (blue solid line), displayed here together for direct comparison. We only consider in this subsection the analysis regarding the cubic distribution of molecules, but the same conclusions stand if the optimized molecular distribution is considered.

**Figure 8:**
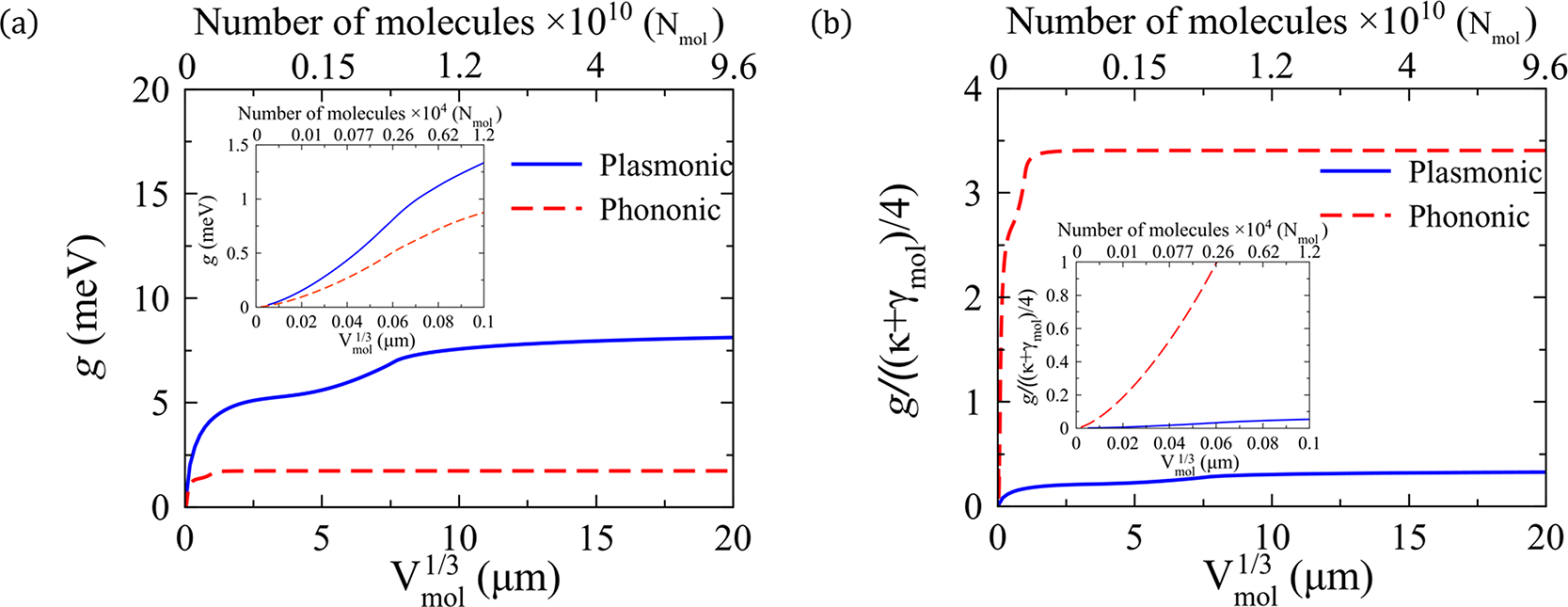
Comparison of the coupling of SiC and Au bowtie nanoantennas with surrounding molecules. The nanogap distance is *d*_g_ = 60 nm in both cases. (a) Collective coupling strength as a function of the cubic root of the cubic volume occupied by the molecules, $V_{\mathrm{mol}}^{1/3}$ (bottom axis) and the corresponding number of molecules, N_mol_ (upper axis). These results correspond to those shown in Figure 7d for a gold plasmonic nanoantenna (blue solid line) and in Figure 3b for a phononic nanoantenna (red dashed line). The inset shows a zoom into the region of small volumes (small number of molecules). (b) Same that in (a), but normalized by $\frac{\kappa +\gamma_{\mathrm{mol}}}{4}$ (with the corresponding κ values **ℏ** *κ*_pl_ = 98.51 meV for plasmonic and **ℏ** *κ*_ph_ = 1.1 meV for phononic nanoantennas). The inset shows this ratio in the region of small volumes (small number of molecules).

We find that *g*_sa_ is always larger for the gold plasmonic nanoantenna than for the SiC phononic counterpart. For example, in the case of a small volume of molecules $V_{\mathrm{mol}}^{1/3}=0.1$ μm, the value of *g*_sa_ obtained for the gold plasmonic nanoantenna is almost 50% larger than that of the phononic nanoantenna (see inset in Figure 8a). For larger volumes, the difference becomes even larger. For example, the value of *g*_sa_ obtained for the gold plasmonic nanoantenna is 460% larger than that of the phononic nanoantenna for $V_{\mathrm{mol}}^{1/3}=15$ μm. However, we emphasize that to observe the phenomena characteristic of strong coupling, we also need to consider the average losses of the system ((κ + γ_mol_)/2). In typical configurations of vibrational strong coupling, the losses of the IR plasmonic mode are much larger than those of the phononic mode and of the molecular vibrations. Thus, as a broad general rule, we obtain that it is easier to reach vibrational strong coupling using phononic rather than noble-metal plasmonic nanoantennas. This trend is illustrated in Figure 8b, which shows the collective coupling strength normalized by $\frac{\kappa +\gamma_{\mathrm{mol}}}{4}$ using the corresponding κ values of the nanoantennas (**ℏ** *κ*_ph_ = 1.1 meV for phononic nanoantennas and **ℏ** *κ*_pl_ = 98.51 meV for gold plasmonic nanoantennas, respectively), for all molecular volumes. The ratio is significantly larger for the phononic nanoantenna, as expected. Nevertheless, if the molecular losses were comparable to the plasmonic losses, then it would be easier to reach strong coupling with noble-metal plasmonic nanoantennas.

We focus next on comparing the maximum collective coupling strength *g*_sa_^max^ between the collective molecular vibrations and the phononic and noble-metal plasmonic bowtie nanoantennas, which is obtained when these nanostructures are fully surrounded by an infinite number of molecules. To this purpose, we consider the difference in how these two types of nanoantennas store the electromagnetic energy. If we consider eq 15 in a situation where the molecules occupy the full volume outside the nanoantennas, the maximum coupling strength obtained is mainly determined by the ratio of the electromagnetic energy stored outside the nanoantenna with respect to the total electromagnetic energy stored in the optical mode, as well as by the properties of the molecules and the dielectric function of the nanoantenna. Equation 15 is valid for small nanoantennas, but the general conclusions should also be applicable to understand the trends of strongly radiative nanoantennas. In particular, in small IR noble-metal plasmonic nanoantennas (shifted thanks to very thin layer structures), where the quasistatic approximation can be applied and which are resonant at large wavelengths, half of the electromagnetic energy is inside the nanoantenna, and half outside,^83^ which at resonance (ω_ph_ = ω_mol_) leads to ≈ *S*/2 (i.e., half the value of the energy splitting in the bulk dispersion of the molecules if losses are ignored).^45^ Interestingly, this *S*/2 maximum is the same as the one obtained in ref 45 for a Fabri-Pérot microcavity, but in this latter case the reason for this result is that half of the energy is stored in the electric field and half of it in the magnetic field. Since most of the electromagnetic energy in phononic nanoantennas is contained within the material, with only a small portion outside, in this type of nanoantennas, *g*_sa_^max^ is considerable weaker compared to gold plasmonic nanoantennas (Figure 4b, where **ℏ** *S* = 12 meV). More details are given in Section S3 of the Supporting Information. Last, we emphasize that the maximum value of the coupling strength *S*/2 that can be obtained with noble-metal plasmonic nanoantennas (eq 18) has been derived using eq 17, which is only valid in the quasistatic limit. Indeed, the results obtained in Figure 7d with the more general semianalytical (eq 14) suggest that strongly radiative noble-metal plasmonic nanoantennas may enable larger coupling strengths, but fully assessing this possibility would require future analysis.

## 4 Summary and Conclusions

We have developed a detailed analysis of the interaction of infrared nanoantenna modes with collective molecular vibrations, and identified the differences between using phononic and noble-metal plasmonic nanoantennas in this context.

Crucially, our approach is based on the use of semianalytical equations to obtain the collective coupling strength in a systematic way. The electromagnetic fields required as input for these equations are calculated from a single simulation of the bare nanoantennas without molecules. As a consequence, only one simulation is enough to obtain the value of the collective coupling strength in all possible molecular distributions. First, we adopt an equation valid for phononic nanoantennas that are sufficiently small to be weakly radiative. Afterward, we modify the equation to study the coupling with large infrared noble-metal plasmonic nanoantennas. These noble-metal plasmonic nanoantennas are characterized by strong radiative losses and thus require an approach beyond the quasistatic approximation, with application of results from quasi-normal mode (QNM) theory. We validate the semianalytical equations by comparing the values of *g* calculated with these semianalytical equations to those obtained by fitting the extinction cross-section spectra using a coupled harmonic oscillator model.

We have focused on the coupling with vibrational collective modes, but the same general approach is applicable to the coupling with other excitations such as phonons or collective excitonic modes. Furthermore, this analysis can be extended to collective coupling with plasmonic resonances in heavily doped semiconductor nanoantennas or with dielectric resonances in nanoantennas made of transparent materials. Future work could also generalize the semianalytical equations so that they can be applied when the high-frequency permittivity *ε*_mol,∞_ of the molecules is not equal to one (or, more generally, to the value of the surrounding medium). We do not expect this generalization to *ε*_mol,∞_ ≠ 1 to affect the general behavior described in the main text, but it will shift the nanoantenna resonances and affect the fraction of energy of the nanoantenna mode that is stored in the volume occupied by the molecules, thus affecting the exact values of the coupling strength.

One of the main advantages of the semianalytical description of the coupling is that it facilitates quantifying the dependence of the coupling strength on both the number of molecules N_mol_ and on their spatial distribution. In particular, we analyze the coupling strength for increasing N_mol_ , focusing on two representative types of molecular distributions: molecules placed inside a cubic volume with center near the field-enhancement hot spot, and an optimal molecular distribution with the molecules placed in the positions that maximize the coupling strength. Our results show the expected fast increase of the coupling strength when one increases the number of molecules in regions of strong near field. We also use these equations to illustrate that small nanogaps are beneficial to couple more efficiently with a small number of molecules, but this advantage is mostly or totally lost in the presence of many molecules.

When comparing the performance of phononic and noble-metal plasmonic nanoantennas, we find that the collective coupling strength is higher when using the plasmonic rather than the phononic bowtie nanoantennas for identical molecular distributions. However, since phononic losses are much lower than plasmonic losses (and typical vibrational losses are comparatively weak), it is easier to reach the strong coupling regime with phononic nanoantennas. Further, it is possible to simplify the equations that describe the coupling when the nanoantennas are fully surrounded by molecules. This analysis indicates that the coupling strength is smaller for phononic than for noble-metal plasmonic nanoantennas largely because the energy is strongly concentrated inside the nanoantennas in the former case. The simplified expressions of the coupling also indicate that the maximum coupling strength in the quasistatic approximation depends on the resonant frequency of the infrared mode, but otherwise not on the shape of the nanoantennas. The analysis presented here thus provides insights into the coupling of infrared plasmonic and phononic nanoantenna modes to collective molecular vibrations supported by arbitrary molecular distributions, which can be useful in the design of nanoantenna architectures aiming at obtaining strong coupling with few molecules.

## Supplementary Material



## Data Availability

The data that support the findings of this study can be found at https://digital.csic.es/handle/10261/393053.

## Notes

Fitting of simulated spectra and value of fit parameters within the coupled harmonic oscillator model; derivation of the semianalytical equation of the collective coupling strength; derivation of the analytical equation of the collective coupling strength when the nanoantenna is fully surrounded by molecules; derivation of the energy density for Au plasmonic nanoantennas; procedure to remove the radiative contribution to the emitted fields; further analysis of the semianalytical expression developed for weakly radiative nanoantennas when analyzing plasmonic nanoantennas; and systematic analysis of the collective coupling strength of a bowtie nanoantenna with corner radius *r* = 30 nm (PDF)
