# Supplementary material for: Semianalytical Treatment of Collective Vibrational Strong Coupling in Infrared Phononic and Plasmonic Nanoantennas
Source: J Phys Chem C Nanomater Interfaces. 2025 Jun 27;129(27):12374–90. doi: 10.1021/acs.jpcc.5c00458 (PMC12512038; doi:10.1021/acs.jpcc.5c00458)
Supplement: Supplementary file 1 [file jp5c00458_si_001.pdf]

Supporting information for Semi-analytical treatment of collective vibrational strong coupling in infrared phononic and plasmonic nanoantennas

Jonathan Sepúlveda,[†,‡] José Luis Montaño-Priede,[†] Javier Aizpurua,[*,¶,§,‡] and Rubén Esteban[*,†,¶]

†*Centro de Física de Materiales (CFM-MPC), CSIC-UPV/EHU, Paseo Manuel Lardizabal 5, Donostia-San Sebastián 20018, Spain*

‡*Department of Electricity and Electronics, FCT-ZTF, UPV/EHU, Leioa 48940, Spain*

¶*Donostia International Physics Center (DIPC), Paseo Manuel Lardizabal 4, 20018 Donostia-San Sebastián, Spain*

§*IKERBASQUE, Basque Foundation for Science, Plaza Euskadi 5, 48009 Bilbao, Spain*

E-mail: aizpurua@ehu.eus; ruben.esteban@ehu.eus

Contents

S1 Fitting of simulated spectra and value of fit parameters within the coupled harmonic oscillator model **S3**

S2 Derivation of the semi-analytical equation of the collective coupling strength **S6**

S3 Derivation of the analytical equation of the collective coupling strength when the nanoantenna is fully surrounded by molecules **S11**

S4 Derivation of the energy density for Au plasmonic nanoantennas **S15**

S5 Procedure to remove the radiative contribution to the emitted fields **S17**

S6 Further analysis of the semi-analytical expression developed for weakly radiative nanoantennas when analyzing plasmonic nanoantennas **S20**

S7 Systematic analysis of the collective coupling strength of a bowtie nanoantenna with corner radius $r = 30$ nm **S21**

References **S24**

S1 Fitting of simulated spectra and value of fit parameters within the coupled harmonic oscillator model

In the main text, one of the approaches that we use to determine the collective coupling strength between infrared nanoantenna modes and molecular vibrations is to use a coupled harmonic oscillator model to fit the simulated extinction cross-section spectra of the system. Here, we present the fittings not shown in the main text and summarize the values of the parameters used in all the fittings.

We first consider the SiC bowtie nanoantenna spectra presented in Section 3.1 of the main text, which is coupled to different cubic distributions of molecules. To fit the extinction cross-section, we fix the following values $\hbar\omega_{\mathrm{ph}} = 0.106$ eV, $\hbar\kappa_{\mathrm{ph}} = 1.1$ meV, and $\hbar\gamma_{\mathrm{m}} = \hbar\gamma_{\mathrm{m}'} = 0.94$ meV, while the fitting parameters are g_{ho}, ω_{m}, $\omega_{\mathrm{m}'}$ $\mathbf{F}_{\mathrm{ph}}$, and $\mathbf{F}_{\mathrm{m}'}$. The values of these parameters extracted from the fits are summarized in Table S1, where the first column gives the volume occupied by each cubic distribution of molecules considered. The simulated extinction cross-section spectrum of the SiC bowtie nanoantenna coupled to the three molecular distributions occupying $\mathrm{V}_{\mathrm{mol}} \approx 0.001\ \mu\mathrm{m}^3$, $\mathrm{V}_{\mathrm{mol}} \approx 1\ \mu\mathrm{m}^3$, and $\mathrm{V}_{\mathrm{mol}} \approx 8\ \mu\mathrm{m}^3$ are plotted in Figure S1 (blue solid line) together with the result of the fits obtained using the values in Table S1 (red dashed line). The spectra are plotted as a function of the photon energy (bottom axis) and the wavenumber (upper axis), and the corresponding results for $\mathrm{V}_{\mathrm{mol}} \approx 0.125\ \mu\mathrm{m}^3$ are plotted in Figure 2b in the main text.

Table S1: Fitting parameters obtained from the fits of the extinction cross-section spectra of the SiC bowtie nanoantenna coupled to different cubic distribution of molecules. Column 1: Volume occupied by the molecules V_{mol}. Columns 2-6: Fitting parameters used to obtain the fits in Figure S1 and in Figure 2b in the main text.

V_{mol} (μm^3)	$\hbar\omega_m$ (eV)	$\hbar\omega_{m'}$ (eV)	$\mathbf{F}_{ph}$ $\times 10^{-8}$	$\mathbf{F}_{m'}$ $\times 10^{-8}$	$\hbar g_{ho}$ (meV)
$\approx$0.001	1.058	0	5	0	0.86
$\approx$0.125	0.1056	0.106	8	3.5	1.39
$\approx$1	0.106	0.106	3.7	3	1.64
$\approx$8	1.058	0.106	9.3	8.5	1.72

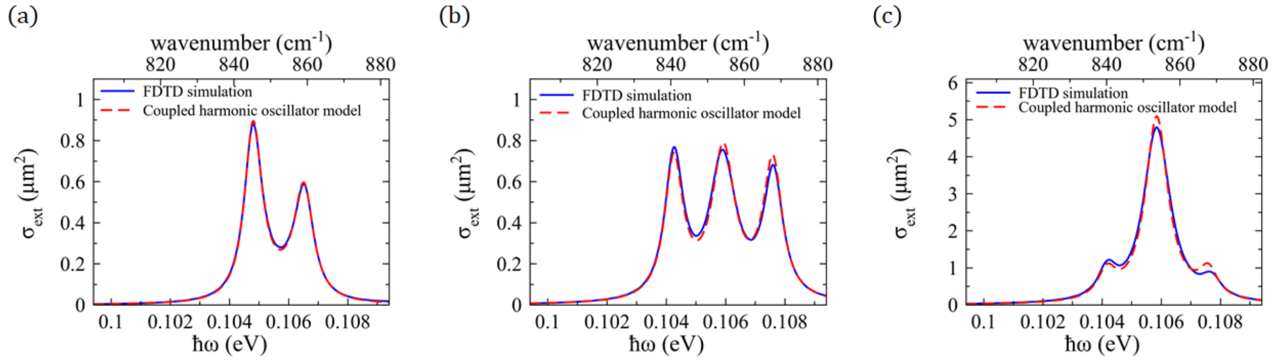

Figure S1: Fits of the infrared response of the phononic (SiC) bowtie nanoantenna with nanogap distance of d_g = 60 nm. (a) Infrared response of the SiC bowtie nanoantenna coupled to a cubic distribution of molecules of volume $V_{mol} \approx 0.001$ μm^3 and illuminated by a plane wave polarized along the bowtie axis (x axis) and propagating in the $-z$ direction normal to the nanoantenna horizontal surface (see coordinate axis in Figure 1b of the main text). The blue solid line corresponds to the simulated extinction cross-section spectra plotted as a function of the photon energy (bottom axis) and the wavenumber (upper axis). The red dashed line corresponds to the results of the fit obtained with the coupled harmonic oscillator model (eq 4 in the main text), and the fitting values in Table S1. (b) Same as in (a) but using a cubic distribution of $V_{mol} \approx 1$ μm^3. (c) Same as in (a) but using a cubic distribution of $V_{mol} \approx 8$ μm^3.

We next consider the coupling of the gold plasmonic bowtie nanoantenna with molecules occupying a cubic region of volume V_{mol}. The value of the fixed parameters are $\hbar\omega_{pl} = 0.106$ meV, $\hbar\kappa_{pl} = 98.51$ meV, the vibrational losses are $\hbar\gamma_m = \hbar\gamma_{m'} = 0.94$ meV, and the fitting parameters are g_{ho}, ω_m, $\omega_{m'}$ $\mathbf{F}_{pl}$, and $\mathbf{F}_{m'}$. We plot in Figure S2 the extinction cross-section spectrum of the system (blue solid line) as a function of the photon energy (bottom axis) and the wavenumber (upper axis), together with the fits obtained from the coupled harmonic

oscillator model (red dashed line). These results are obtained for $V_{mol} \approx 0.125$ μm^3, and $V_{mol} \approx 125$ μm^3. The values of the fitting parameters used for these fits in Figure S2 and the fit in Figure 5c for $V_{mol} \approx 1$ μm^3 are summarized in Table S2.

Table S2: Fitting parameters obtained from the fits of the extinction cross-section spectra of the Au plasmonic bowtie nanoantenna coupled to different cubic distribution of molecules. Column 1: Volume occupied by the molecules V_{mol}. Columns 2-6: Fitting parameters used to obtain the fits in Figure S2 and in Figure 5c in the main text.

V_{mol} (μm^3)	$\hbar\omega_m$ (eV)	$\hbar\omega_{m'}$ (eV)	$F_{pl} \times 10^{-6}$	$F_{m'}$	$\hbar g_{ho}$ (meV)
≈0.125	1.059	0.106	3.5	4.2×10^{-12}	3.15
≈1	0.106	0.106	3.4	4.2×10^{-12}	3.99
≈125	0.1063	0.1054	3.5	3.3×10^{-7}	5.28

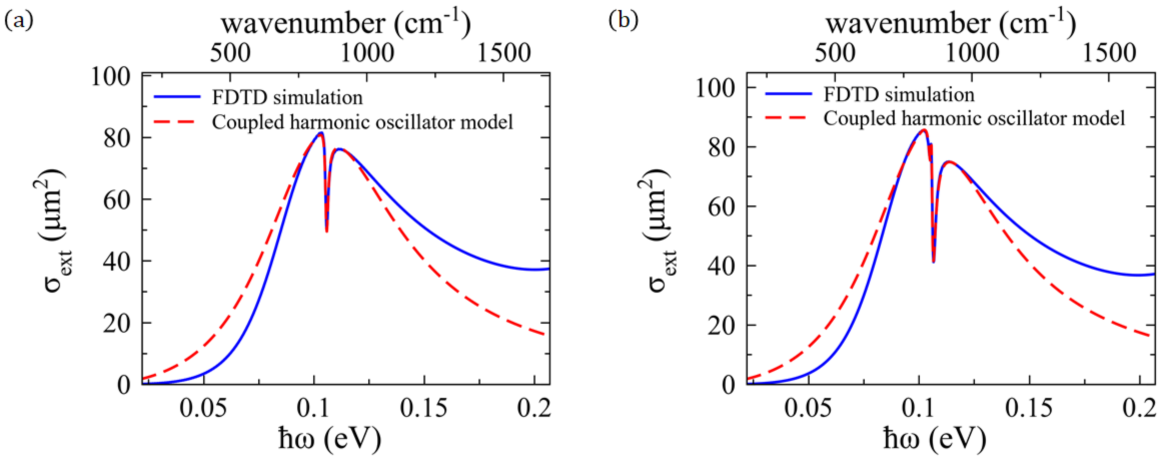

Figure S2: Fits of the infrared response of the plasmonic (Au) bowtie nanoantenna with nanogap distance of $d_g = 60$ nm. (a) Infrared response of the Au bowtie nanoantenna coupled to a cubic distribution of molecules of volume $V_{mol} \approx 0.125$ μm^3 and illuminated by a plane wave polarized along the bowtie axis (x axis) and propagating in the $-z$ direction normal to the nanoantenna horizontal surface (see coordinate axis in Figure 5a of the main text). The blue solid line corresponds to the simulated extinction cross-section spectra plotted as a function of the photon energy (bottom axis) and the wavenumber (upper axis). The red dashed line corresponds to the results of the fit obtained with the coupled harmonic oscillator model (eq 4 in the main text) and the fitting values in Table S2. (b) Same that in (a) but using a cubic distribution of $V_{mol} \approx 125$ μm^3.

S2 Derivation of the semi-analytical equation of the collective coupling strength

In the main text, we show that the collective coupling strength can be analyzed systematically using eq 12, which only requires simulating the fields induced by the bare nanoantenna in the absence of molecules. The advantage of this methodology is that it only requires a single simulation to perform a systematic study of the coupling of nanoantennas with an arbitrary large set of molecular distributions. In this supplementary section, we derive this equation in more detail, following a similar procedure as the one discussed in Ref. 1, which analyzes the coupling between molecular vibrations and optical modes in dielectric resonators. Similar derivations can be found in Refs. 2–5. Here, we focus on the (small) phononic nanoantennas because they can be treated within the quasistatic approximation[6], but the equations are also useful to treat the large gold plasmonic nanoantennas after implementing the modifications discussed in the main text.

In a Cavity Quantum Electrodynamics description, we can define the transition dipole moment operator associated with the vibrational response of an isotropic molecule j as[7,8]

$$\hat{\boldsymbol{\mu}}_j = \mu \left[\left(\hat{b}_{x,j} + \hat{b}^{\dagger}_{x,j} \right) \mathbf{n}_x + \left(\hat{b}_{y,j} + \hat{b}^{\dagger}_{y,j} \right) \mathbf{n}_y + \left(\hat{b}_{z,j} + \hat{b}^{\dagger}_{z,j} \right) \mathbf{n}_z \right] \tag{S1}$$

where $\hat{b}_{q,j}$ and $\hat{b}^{\dagger}_{q,j}$ correspond to the annihilation and creation operators of a harmonic oscillator that represent a vibrational mode along q, with $q = x, y, z$, $\mathbf{n}_q$ is the unitary vector in this direction and μ corresponds to the transition dipole moment, which is considered to be the same for all molecules. Thus, at this stage, we describe the isotropic response through three identical orthogonal vibrations (same strength, (angular) frequency ω_{mol} and losses γ_{mol}). μ can be obtained by relating this quantity with the dielectric function of an isotropic ensemble of these molecules $\varepsilon_{\mathrm{mol}}(\omega)$ (eq 2 in the main text)[Sa]. We do this through

[Sa] In principle, the resonant frequency and losses of $\varepsilon_{\mathrm{mol}}(\omega)$ are not the same as the corresponding values of the individual molecules due to the direct coupling between molecules. However, for most systems, it is

the polarization vector $\mathbf{P}(\mathbf{r}_j)$ at the position of the molecule $\mathbf{r}_j$, which is connected with $\varepsilon_{\mathrm{mol}}(\omega)$ by

$$\mathbf{P}(\mathbf{r}_j) = \varepsilon_0 \left(\varepsilon_{\mathrm{mol}}(\omega) - 1\right) \mathbf{E}(\mathbf{r}_j). \tag{S2}$$

Additionally, the induced dipole of each molecule is given by $\mathbf{p}_j = \alpha(\omega)\,\mathbf{E}(\mathbf{r}_j)$, where $\alpha(\omega)$ is the isotropic classical polarizability of each molecule. Assuming that the effective volume per molecule is V_μ, we can also express the polarization vector as

$$\mathbf{P}(\mathbf{r}_j) = \frac{\alpha(\omega)}{\mathrm{V}_\mu} \mathbf{E}(\mathbf{r}_j). \tag{S3}$$

Replacing eq (S2) into eq (S3), we obtain the relationship between the dielectric function of the molecules and the classical polarizability of each molecule

$$\alpha(\omega) = \varepsilon_0 \left(\varepsilon_{\mathrm{mol}}(\omega) - 1\right) \mathrm{V}_\mu. \tag{S4}$$

Furthermore, the polarizability and the transition dipole moment of each molecule are related via[10]

$$\alpha(\omega) = \frac{|\mu|^2}{\hbar} \frac{2\omega_{\mathrm{mol}}}{\omega_{\mathrm{mol}}^2 - \omega^2 - i\gamma_{\mathrm{mol}}\omega}, \tag{S5}$$

where γ_{mol} is the damping rate of the molecular vibration, and ω_{mol} the frequency of the vibrational mode. Using eq S4, eq S5 and eq 2 of the main text, we obtain the expression of the transition dipole moment

$$\mu = \sqrt{\frac{\hbar \varepsilon_0 \mathrm{V}_\mu S^2}{2\omega_{\mathrm{mol}}}}, \tag{S6}$$

where S is the strength of the oscillator (given in eq 2 in the main text).

possible to neglect this direct coupling by renormalizing the molecular frequency and losses so that they take the same value as in the expression of $\varepsilon_{\mathrm{mol}}(\omega)$.[9] We follow this approach here.

Similarly, the electric field operator of the phononic mode is quantized according to[11]

$$\hat{\mathbf{E}}\left(\mathbf{r}_j\right)=\sqrt{\frac{\hbar\omega_{\mathrm{ph}}}{2\varepsilon_0 \mathrm{V}_{\mathrm{qst}}^{\mathrm{eff}}}}\left(\frac{\mathbf{E}^s\left(\mathbf{r}_j\right)}{\left|\mathbf{E}_{\max}^s\right|}\hat{a}+\frac{\mathbf{E}^{s^*}\left(\mathbf{r}_j\right)}{\left|\mathbf{E}_{\max}^s\right|}\hat{a}^{\dagger}\right), \tag{S7}$$

where ε_0 is the vacuum permittivity, ω_{ph} is the (angular) frequency of the phononic mode, $\hat{a}$ and $\hat{a}^{\dagger}$ are the annihilation and creation operators associated with this mode, respectively, and $\mathrm{V}_{\mathrm{qst}}^{\mathrm{eff}}$ is the effective mode volume (obtained following the procedure explained in the main text). Further, $\mathbf{E}_{\max}^s$ is the maximum amplitude of the scattered electric field, $\mathbf{E}^s\left(\mathbf{r}_j\right)$ the scattered electric field at the position of the molecule j, and the superindex $*$ indicates the complex conjugate. The electric fields can be obtained from a simulation of the bare nanoantenna (i.e. without molecules) by exciting the bowtie with a plane wave of frequency ω_{ph} polarized along the bowtie axis and propagating in the $-z$ direction perpendicular to the nanoantenna horizontal surfaces (see axis in Figure 1b in the main text). The scattered fields are obtained by subtracting to the total fields those of the incoming plane wave illumination. This approach assumes that the optical response at ω_{ph} is dominated by a single mode.

We next write the Hamiltonian of the system in the case of a single molecular vibration j as

$$\hat{H}=\hat{H}_{\mathrm{ph}}+\hat{H}_{\mathrm{mol}}+\hat{H}_{\mathrm{int}}, \tag{S8}$$

where

$$\hat{H}_{\mathrm{ph}}=\hbar\omega_{\mathrm{ph}}\hat{a}^{\dagger}\hat{a}, \tag{S9}$$

$$\hat{H}_{\mathrm{mol}}=\hbar\omega_{\mathrm{mol}}\left(\hat{b}_{x,j}^{\dagger}\hat{b}_{x,j}+\hat{b}_{y,j}^{\dagger}\hat{b}_{y,j}+\hat{b}_{z,j}^{\dagger}\hat{b}_{z,j}\right), \tag{S10}$$

$$\hat{H}_{\mathrm{int}}=-\hat{\boldsymbol{\mu}}_j\cdot\hat{\mathbf{E}}\left(\mathbf{r}_j\right), \tag{S11}$$

and $\hat{H}_{\mathrm{ph}}$, $\hat{H}_{\mathrm{mol}}$ and $\hat{H}_{\mathrm{int}}$ are the Hamiltonians of the dipolar phononic mode of the nanoantenna, of the molecular vibration, and of the molecule-nanoantenna interaction, respectively. Replacing

eq S1 and eq S7 into eq S11, the interaction Hamiltonian in the rotating wave approximation is written as

$$\hat{H}_{\mathrm{int}} = \hbar \left(\mathrm{g}_x \hat{b}^\dagger_{x,j} \hat{a} + \mathrm{g}^*_x \hat{b}_{x,j} \hat{a}^\dagger + \mathrm{g}_y \hat{b}^\dagger_{y,j} \hat{a} + \mathrm{g}^*_y \hat{b}_{y,j} \hat{a}^\dagger + \mathrm{g}_z \hat{b}^\dagger_{z,j} \hat{a} + \mathrm{g}^*_z \hat{b}_{z,j} \hat{a}^\dagger \right), \tag{S12}$$

where

$$\mathrm{g}_q = -\sqrt{\frac{\omega_{\mathrm{ph}} \mathrm{V}_\mu S^2}{4\omega_{\mathrm{mol}} \mathrm{V}^{\mathrm{eff}}_{\mathrm{qst}}}} \frac{E^s_q(\mathbf{r}_j)}{|\mathbf{E}^s_{\mathrm{max}}|}, \tag{S13}$$

and $q = x, y, z$.

We define next the operator

$$\hat{b}_j = \beta \left(\mathrm{g}^*_x \hat{b}_{x,j} + \mathrm{g}^*_y \hat{b}_{y,j} + \mathrm{g}^*_z \hat{b}_{z,j} \right), \tag{S14}$$

which represents the vibrational mode of the molecule j that couples with the phononic mode. Here, $\beta = 1/\sqrt{|\mathrm{g}_x|^2 + |\mathrm{g}_y|^2 + |\mathrm{g}_z|^2}$ is a normalization constant chosen to satisfy the commutation relation $[\hat{b}_j, \hat{b}^\dagger_j] = 1$. Expressing the interaction Hamiltonian in this basis, we obtain

$$\hat{H}_{\mathrm{int}} = \hbar \mathrm{g}^{(j)}_{\mathrm{sa}} \left(\hat{b}_j \hat{a}^\dagger + \hat{b}^\dagger_j \hat{a} \right), \tag{S15}$$

with

$$\mathrm{g}^{(j)}_{\mathrm{sa}} = \sqrt{\frac{\omega_{\mathrm{ph}} \mathrm{V}_\mu S^2}{4\omega_{\mathrm{mol}} \mathrm{V}^{\mathrm{eff}}_{\mathrm{qst}}}} \frac{|\mathbf{E}^s(\mathbf{r}_j)|}{|\mathbf{E}^s_{\mathrm{max}}|}, \tag{S16}$$

the coupling strength between the phononic nanoantenna and molecule j. In this equation, $|\mathbf{E}^s(\mathbf{r}_j)| = \sqrt{|E^s_x(\mathbf{r}_j)|^2 + |E^s_y(\mathbf{r}_j)|^2 + |E^s_z(\mathbf{r}_j)|^2}$ is the amplitude of the scattered electric field at the position of the molecule j. Furthermore, to transform the molecular Hamiltonian in eq S10, we also need to define two additional vibrational modes ($\hat{b}'_j$, $\hat{b}''_j$) orthogonal to $\hat{b}_j$ and that do not couple with the phononic mode. In this case, eq S10 transforms into $\hat{H}_{\mathrm{mol}} =$

$\hbar\omega_{\text{mol}}\left(\left(\hat{b}_j\right)^\dagger\hat{b}_j + \left(\hat{b}'_j\right)^\dagger\hat{b}'_j + \left(\hat{b}''_j\right)^\dagger\hat{b}''_j\right)$. These additional vibrations do not contribute to the coupling and are ignored in the following (they could, however, contribute to the central peak in Figure 2b). Next, we can write the Hamiltonian involving all N_{mol} molecules,

$$\hat{H} = \hbar\omega_{\text{ph}}\hat{a}^\dagger\hat{a} + \hbar\omega_{\text{mol}}\sum_{j=1}^{\text{N}_{\text{mol}}}\hat{b}_j^\dagger\hat{b}_j + \sum_{j=1}^{\text{N}_{\text{mol}}}\hbar\text{g}_{\text{sa}}^{(j)}\left(\hat{b}_j\hat{a}^\dagger + \hat{b}_j^\dagger\hat{a}\right), \tag{S17}$$

which corresponds to eq 6 in the main text. Following footnote Sa, we do not include direct molecule-molecule interactions[9] because the effect of this coupling has been incorporated into a renormalization of the frequency ω_{mol}. To obtain the collective coupling strength g_{sa} between a collective vibrational mode that involves the coherent oscillations of all molecules and the phononic mode, we define the collective operators $\hat{B}_m$ according to

$$\hat{B}_m = \sum_{j=1}^{\text{N}_{\text{mol}}} c_{mj}\hat{b}_j \ , \tag{S18}$$

where the coefficients satisfy the orthonormality relation $\sum_j c_{mj}c^*_{m'j} = \delta_{mm'}$, so that the commutation relation $\left[\hat{B}_m, \hat{B}_j\right] = \delta_{mj}$ is fullfilled. In this case, the inverse relationship of eq S18 is $\hat{b}_j = \sum_{m=1}^{\text{N}_{\text{mol}}} c^*_{mj}\hat{B}_m$. Choosing $c_{1j} = \text{g}_{\text{sa}}^{(j)}/\sqrt{\sum_k |\text{g}_{\text{sa}}^{(k)}|^2}$ (for $m = 1$), substituting eq S18 into S17, and exploiting the orthonormality of the c_{mj} coefficients, we obtain the Hamiltonian in the collective mode basis

$$\hat{H} = \hbar\omega_{\text{ph}}\hat{a}^\dagger\hat{a} + \hbar\omega_{\text{mol}}\sum_{m=1}^{\text{N}_{\text{mol}}}\hat{B}_m^\dagger\hat{B}_m + \hbar\left(G_1\hat{B}_1\hat{a}^\dagger + G_1^*\hat{B}_1^\dagger\hat{a}\right), \tag{S19}$$

where $G_1 = G_1^* = \sqrt{\sum_j |\text{g}_{\text{sa}}^{(j)}|^2}$ because of the orthonormal condition. We thus find that the phononic mode only couples with the first collective mode $\hat{B}_1$, the so-called bright mode. The others $\text{N}_{\text{mol}} - 1$ collective operators ($\hat{B}_{m\geq 2}$) correspond to dark collective modes that do not couple with the phononic mode we are considering. The collective coupling strength is then given by

$$\mathrm{g_{sa}} = \sqrt{\sum_{j=1}^{\mathrm{N_{mol}}} \frac{\omega_{\mathrm{ph}} \mathrm{V}_{\mu} S^2}{4\omega_{\mathrm{mol}} \mathrm{V}_{\mathrm{qst}}^{\mathrm{eff}}} \frac{\left|\mathbf{E}^s\left(\mathbf{r}_j\right)\right|^2}{\left|\mathbf{E}_{\mathrm{max}}^s\right|^2}}, \tag{S20}$$

corresponding to eq 11 in the main text. Finally, we assume a continuous distribution of molecules and convert the sum to an integral by multiplying the term in the sum by the molecular density ($1/\mathrm{V}_{\mu}$). We obtain in this way the final expression of the collective coupling strength (eq 12 in the main text)

$$\mathrm{g_{sa}} = \sqrt{\frac{1}{4} \frac{\omega_{\mathrm{ph}} S^2 \int d\mathrm{V}_{\mathrm{mol}} \left|\mathbf{E}^s\left(\mathbf{r}\right)\right|^2}{\omega_{\mathrm{mol}} \left|\mathbf{E}_{max}^s\right|^2 \mathrm{V}_{\mathrm{qst}}^{\mathrm{eff}}}}. \tag{S21}$$

S3 Derivation of the analytical equation of the collective coupling strength when the nanoantenna is fully surrounded by molecules

In this supplementary section, we derive in detail simple expressions of the maximum collective coupling strength between molecules and small nanoantennas, which is found when the molecules occupy all the volume surrounding the nanoantenna. We focus here on the phononic nanoantennas, but the same analysis can be extended to small plasmonic nanoantennas as discussed below. From eq 8 and eq 12 in the main text, we can write

$$\mathrm{g}^2 = \frac{\omega_{\mathrm{ph}} S^2}{2\omega_{\mathrm{mol}}} \mathrm{F}, \tag{S22}$$

where ω_{ph}, and ω_{mol} are the angular frequency of the dipolar phononic mode and the vibrational mode of the molecules, respectively, and S is the strength of the oscillator (see eq 2 in the main text). Last

$$\mathrm{F} = \frac{\frac{1}{2}\varepsilon_0 \int d\mathrm{V}_{\mathrm{mol}} \left|\mathbf{E}^s\left(\mathbf{r}\right)\right|^2}{\frac{1}{2}\int_{\mathrm{total}} d\mathrm{V}_{\mathrm{int}} \left(\varepsilon_0 \left(\mathrm{Re}\left(\varepsilon_r\left(\omega_{\mathrm{ph}}\right)\right) + \frac{2\omega_{\mathrm{ph}}}{\gamma_{\mathrm{SiC}}}\mathrm{Im}\left(\varepsilon_r\left(\omega_{\mathrm{ph}}\right)\right)\right) |\mathbf{E}^s\left(\mathbf{r}\right)|^2 + \mu_0 |\mathbf{H}^s\left(\mathbf{r}\right)|^2\right)}, \tag{S23}$$

is the ratio of the electric energy in the region occupied by the molecules and the total electromagnetic energy of the system. Here, $|\mathbf{E}^s(\mathbf{r})|$, and $|\mathbf{H}^s(\mathbf{r})|$ are the corresponding module of the scattered electric and magnetic fields, respectively. The volume of integration $\mathrm{V}_{\mathrm{mol}}$ in the numerator corresponds to the region filled with molecules, and $\mathrm{V}_{\mathrm{int}}$ in the denominator corresponds to the full (infinite) space, including the inside and outside the nanoantenna. The dielectric function in the region of the nanoantenna is $\varepsilon_r = \varepsilon_{\mathrm{SiC}}$ and in the outside region $\varepsilon_r = 1$. In the denominator of eq S23, we have used the expression of the energy density inside the phononic material that describes it as proportional to $\mathrm{Re}\left(\varepsilon_r\left(\omega_{\mathrm{ph}}\right)\right) + \frac{2\omega_{\mathrm{ph}}}{\gamma_{\mathrm{SiC}}}\mathrm{Im}\left(\varepsilon_r\left(\omega_{\mathrm{ph}}\right)\right)$ (with γ_{SiC} the losses of the material) to avoid incorrect results near the transverse phononic frequency[12–14] ω_t. In the outside region, this term can just be substituted by $\varepsilon_r = 1$. Importantly, in this approach the molecules only determine the value of S in eq. S22 and the region of integration in the numerator in eq S23, but not the electromagnetic fields in eq S23, which are calculated in the absence of molecules. Furthermore, in the quasistatic approximation, $|\mathbf{H}^s(\mathbf{r})| \approx 0$, so that in the following the denominator in eq S23 only depends on the scattered electric field. We separate next the integral in the denominator of eq S23 into two parts, one corresponding to the region occupied by the nanoantenna and the other to the molecular region. Further, we consider from here that the molecules occupy all the outside volume, so that the integrals in the numerator and the denominator extend over the same region. The result is

$$\mathrm{F} = \frac{\frac{1}{2}\varepsilon_0 \int_{\mathrm{out}} d\mathrm{V}_{\mathrm{mol}} \left|\mathbf{E}^s\left(\mathbf{r}\right)\right|^2}{\frac{1}{2}\varepsilon_0 \int_{\mathrm{in}} d\mathrm{V}_{\mathrm{SiC}} \left(\mathrm{Re}\left(\varepsilon_{\mathrm{SiC}}\left(\omega_{\mathrm{ph}}\right)\right) + \frac{2\omega_{\mathrm{ph}}}{\gamma_{\mathrm{SiC}}}\mathrm{Im}\left(\varepsilon_{\mathrm{SiC}}\left(\omega_{\mathrm{ph}}\right)\right)\right) |\mathbf{E}^s\left(\mathbf{r}\right)|^2 + \frac{1}{2}\varepsilon_0 \int_{\mathrm{out}} d\mathrm{V}_{\mathrm{mol}} |\mathbf{E}^s\left(\mathbf{r}\right)|^2}, \tag{S24}$$

where we have added in the integral the subindex 'in' to indicate the region of volume V_{SiC} occupied by the nanoantenna and the subindex 'out' to indicate the molecular region of volume V_{mol} outside the nanoantenna.

We then relate the volume integral outside and inside the nanoantenna by using the identity valid in the quasistatic regime[15]

$$\int_{\text{out}} d\text{V}_{\text{mol}}\varepsilon_0 |\mathbf{E}^s(\mathbf{r})|^2 = -\int_{\text{in}} d\text{V}_{\text{SiC}}\varepsilon_0 \text{Re}\left(\varepsilon_{\text{SiC}}(\omega_{\text{ph}})\right)|\mathbf{E}^s(\mathbf{r})|^2, \tag{S25}$$

obtaining

$$\text{F} = \frac{-\text{Re}\left(\varepsilon_{\text{SiC}}(\omega_{\text{ph}})\right)}{\frac{2\omega_{\text{ph}}}{\gamma_{\text{SiC}}}\text{Im}\left(\varepsilon_{\text{SiC}}(\omega_{\text{ph}})\right)}. \tag{S26}$$

Substituting eq 1 in the main text into eq S26, we obtain

$$\text{F} = -\frac{\left\{\left(\omega_t^2 - \omega_{\text{ph}}^2\right)\left(\omega_l^2 - \omega_{\text{ph}}^2\right)\right\}}{2\left(\omega_l^2 - \omega_t^2\right)\omega_{\text{ph}}^2}, \tag{S27}$$

where we have considered that $\gamma_{\text{SiC}} \ll \omega_{\text{ph}}$. We next Substitute eq S27 into eq S22 which yields eq 16 in the main text

$$\text{g}_{\text{sa}}^{\text{max}} = \sqrt{-\frac{\omega_{\text{ph}}S^2}{4\omega_{\text{mol}}}\frac{\left\{\left(\omega_t^2 - \omega_{\text{ph}}^2\right)\left(\omega_l^2 - \omega_{\text{ph}}^2\right)\right\}}{\left(\omega_l^2 - \omega_t^2\right)\omega_{\text{ph}}^2}}. \tag{S28}$$

Equation S28 corresponds to the maximum coupling for a given nanoantenna resonant at ω_{ph}. Next, the maximum coupling strength for any possible nanoantenna mode is determined by taking the first derivative of eq S27 with respect to ω_{ph} and equalizing it to zero

$$\frac{d\text{F}}{d\omega_{\text{ph}}} = -\frac{\omega_{\text{ph}}^4 - \omega_t^2\omega_l^2}{\left(\omega_l^2 - \omega_t^2\right)\omega_{\text{ph}}^3} = 0, \tag{S29}$$

which indicates that the maximum for any nanoantenna mode is obtained when the phononic nanoantenna is resonant at frequency $\omega_{\text{ph}} = \sqrt{\omega_t\omega_l}$. Substituting this value in eq S28, we obtain (in resonance, $\omega_{\text{mol}} = \omega_{\text{ph}}$)

$$\mathrm{g}_{\mathrm{sa}}^{\mathrm{max}}\big|_{\omega_{\mathrm{ph}}=\sqrt{\omega_t\omega_l}} = \frac{S}{2}\sqrt{\frac{\omega_l-\omega_t}{\omega_l+\omega_t}}, \tag{S30}$$

the maximum coupling strength between the phononic mode and the collective vibrational mode that can be achieved in resonance for any phononic nanoantenna in the quasistatic approximation.

To obtain the maximum coupling strength for a small plasmonic gold nanoantenna, resonant at infrared frequencies under the quasistatic approximation, we simply use the dielectric function of gold (eq 17 in the main text) instead of that of the phononic material. This change is equivalent to replacing ω_t, ω_l, and γ_{SiC} with the equivalent plasmonic values, $\omega_t \to 0$, $\gamma_{\mathrm{SiC}} \to \gamma_{\mathrm{Au}}$ and $\omega_l \to \omega_{\mathrm{p,Au}}/\sqrt{\varepsilon_{\mathrm{Au},\infty}}$, and it is further justified in Section S4. We then obtain

$$\mathrm{F} = \frac{\omega_{\mathrm{p,Au}}^2 - \varepsilon_{\mathrm{Au},\infty}\omega_{\mathrm{pl}}^2}{2\omega_{\mathrm{p,Au}}^2}, \tag{S31}$$

and

$$\mathrm{g}_{\mathrm{sa}}^{\mathrm{max}} = \sqrt{\frac{\omega_{\mathrm{pl}}S^2}{\omega_{\mathrm{mol}}}\frac{\omega_{\mathrm{p,Au}}^2 - \varepsilon_{\mathrm{Au},\infty}\omega_{\mathrm{pl}}^2}{4\omega_{\mathrm{p,Au}}^2}}, \tag{S32}$$

for a small gold plasmonic nanoantenna with $\gamma_{\mathrm{Au}} \ll \omega_{\mathrm{pl}}$ (note that, strictly, this approximation is not fullfilled in the infrared, but we ignore this here because the coupling strength is typically not affected by the losses). At mid-infrared frequencies $\omega_{\mathrm{pl}} \ll \omega_{\mathrm{p,Au}}$, and thus (in resonance $\omega_{\mathrm{pl}} = \omega_{\mathrm{mol}}$)

$$\mathrm{g}_{\mathrm{sa}}^{\mathrm{max}} \approx \frac{S}{2}. \tag{S33}$$

S4 Derivation of the energy density for Au plasmonic nanoantennas

In the main text, we obtain the electric energy density $\mathrm{u_E}$ at mid-infrared frequency ω and at a point inside a phononic or metallic material of dielectric function $\varepsilon_{\mathrm{mat}}$ from the equation

$$\mathrm{u_E} = \frac{1}{2}\varepsilon_0 \left[\mathrm{Re}(\varepsilon_{\mathrm{mat}}(\omega)) + \frac{2\omega}{\gamma_{\mathrm{mat}}}\mathrm{Im}(\varepsilon_{\mathrm{mat}}(\omega))\right] |E|^2, \tag{S34}$$

where γ_{mat} is the damping rate (γ_{SiC} and γ_{Au} for SiC and Au, respectively) and E is the local field[Sb]. For simplicity, in this section we do not indicate the position $\mathbf{r}$ and we consider the electric field along a fixed direction so that it can be treated as a scalar. Eq. S34 has been derived for phononic materials in Refs. 12,14, but we have used this equation also for Au because the frequency dependence of the Drude dielectric function of a metallic material in the mid-infrared (eq 17) is formally the same as that of a phononic material with a single phonon and $\omega_t = 0$ (eq 1 in the main text). Here, we describe an alternative derivation of eq. S34 for Au (or other metals) at low frequencies. We do not attempt to be rigorous in this derivation, but to sketch an intuitive picture based on circuit theory.

As we are considering the response at small energies, we can use a Drude model description of the Au conductivity σ_{Au}, which is given by

$$\sigma_{\mathrm{Au}} = i\varepsilon_0 \frac{\omega^2_{\mathrm{p,Au}}}{\omega + i\gamma_{\mathrm{Au}}}, \tag{S35}$$

and can be obtained from the Drude dielectric function $\varepsilon_{\mathrm{Au}}(\omega)$ (eq 17 in the main text) and the following relationship between the conductivity and the dielectric function

$$\varepsilon_{\mathrm{Au}}(\omega) = \varepsilon_{\mathrm{Au},\infty} + \frac{i\sigma_{\mathrm{Au}}}{\varepsilon_0\omega}. \tag{S36}$$

[Sb]In equation 18 in the main text, E^2 is used instead of $|E|^2$, but we focus here on the energy density.

In these equations, $\varepsilon_{\mathrm{Au},\infty}$ is the dielectric constant at high frequency and $\omega_{\mathrm{p,Au}}$ the plasma frequency of gold.

On the other hand, we consider gold as a material of complex-valued impedance Z, so that an alternating current (AC) denoted I generates a difference of potential V according to

$$Z = \frac{V}{I}. \tag{S37}$$

If we consider a Au region of length l and transverse area A (small enough so that the fields can be considered constant), we can use the relationships $V = El$, $I = JA$, $J = \sigma_{\mathrm{Au}}E$, where E is again the field and J the current density. We can then write the impedance as

$$Z = \frac{l\gamma_{\mathrm{Au}}}{\varepsilon_0 \omega_{\mathrm{p,Au}}^2 A} - \frac{il\omega}{\varepsilon_0 \omega_{\mathrm{p,Au}}^2 A}. \tag{S38}$$

From the imaginary part of the impedance (reactance, $\chi_L = -\mathrm{Im}(Z)$, notice the different sign convention from that in electronics[16]), we can define the inductance L of a metallic nanoantenna as

$$\chi_L = \omega L = \frac{\omega l}{\varepsilon_0 \omega_{\mathrm{p,Au}}^2 A}, \tag{S39}$$

where

$$L = \frac{l}{\varepsilon_0 \omega_{\mathrm{p,Au}}^2 A}. \tag{S40}$$

The energy stored in this inductance is

$$W = \frac{1}{2} L I^2, \tag{S41}$$

so that replacing eq S40 into S41 and using $I = JA = A\sigma_{\mathrm{Au}}E$ and eq S35, we obtain

$$W = \frac{1}{2}\varepsilon_0 v \left(\frac{\omega_{\mathrm{p,Au}}^2}{\omega^2 + \gamma_{\mathrm{Au}}^2} \right) |E|^2, \tag{S42}$$

where $v = lA$ is the volume of the gold region. Additionally, for the Drude dielectric function in eq 17 in the main text, the following expression

$$\frac{\omega_{\mathrm{p,Au}}^2}{\omega^2 + \gamma_{\mathrm{mat}}^2} = \mathrm{Re}(\varepsilon_{\mathrm{Au}}(\omega)) + \frac{2\omega}{\gamma_{\mathrm{Au}}}\mathrm{Im}(\varepsilon_{\mathrm{Au}}(\omega)). \tag{S43}$$

is verified at small ω. Finally, replacing eq S43 into eq S42, and dividing the resulting equation by the volume v (to relate the energy stored with the energy density, $W = \mathrm{u_E} v$), we recover eq S34, as desired. We emphasize that this expression has been derived for mid-infrared frequencies. At higher energies, the approximate expression $\frac{d(\omega\varepsilon_{\mathrm{mat}}(\omega))}{d\omega}$ can be more appropriate.

S5 Procedure to remove the radiative contribution to the emitted fields

In the main text, we discuss that the direct application of eq 12 does not give the correct results when the volume occupied by the molecules $\mathrm{V_{mol}}$ increases, due to the radiated energy. We also explain how to correct these results for weakly radiative nanoantennas that can be treated within the quasistatic approximation. In this supplementary section, we show how we overcome this difficulty for highly radiative nanoantennas. The main idea is to describe the nanoantenna as an ensemble of point dipoles, whose far field radiation can be calculated and subtracted in a straightforward way. To do this, we first define a rectangular 3D grid of $n \times n \times 1$ cells for each of the two prisms that define the bowtie nanoantenna, as illustrated in Figure S3a-b for two different values of n. The grid is plotted at the horizontal plane $z = 0$ (see axis in Figure 5a in the main text, with $x = y = z = 0$ the center of the nanogap). We calculate the dipole moment of each point dipole in the 3D grid by using

$$\mathbf{p}_j = \int d\mathrm{V}_j \mathbf{P}. \tag{S44}$$

Here, $\mathbf{P}(\mathbf{r}) = \varepsilon_0(\varepsilon_r - 1)\mathbf{E}(\mathbf{r})$ is the polarization vector, where ε_r corresponds to the dielectric function of the nanoantenna in the region occupied by the nanoantenna (gold in our case, $\varepsilon_r = \varepsilon_{\mathrm{Au}}$) and $\varepsilon_r = 1$ outside the nanoantenna. Thus, $\mathbf{P}(\mathbf{r}) = 0$ in the outside region. Furthermore, the subindex j denotes the $j-$th cell in the 3D grid and V_j the volume of the cell. These dipoles are then placed at a position calculated as

$$\mathbf{r}_j = \frac{\int d\mathrm{V}_j|\mathbf{P}| \cdot \mathbf{r}}{\int d\mathrm{V}_j|\mathbf{P}|}, \tag{S45}$$

and marked by asterisks in Figure S3a-b. With this procedure, each point dipole is located within its respective cell, and cells fully located outside the nanoantenna do not contribute because their dipole moments are zero.

Once $\mathbf{r}_j$, and $\mathbf{p}_j$ are known, we calculate the radiative contribution of these dipoles by using[10,16]

$$\mathbf{E}^{\mathrm{dip}}(\mathbf{r}) = \sum_{j=1}^{\mathrm{N_{cell}}} \frac{\mathrm{k_{ph}}}{4\pi\varepsilon_0} \left((\mathbf{n} \times \mathbf{p}_j) \times \mathbf{n} \frac{e^{i\mathrm{k_{ph}} r_{\mathrm{dip}}^{(j)}}}{r_{\mathrm{dip}}^{(j)}} \right). \tag{S46}$$

Here, $\mathrm{k_{ph}}$ corresponds to the vacuum wavenumber of the emitted photons at frequency ω_{ph}, $\mathrm{k_{ph}} = \omega_{\mathrm{ph}}/c_0$, with c_0 the speed of light in the surrounding vacuum. $\mathbf{n}$ is a unitary vector that specifies a direction in the x, y, and z axis, and $r_{\mathrm{dip}}^{(j)} = |\mathbf{r} - \mathbf{r}_j|$ corresponds to the distance between the $j-$th dipole and the observation point.

The radiative contribution given by eq S46 for the two different dipole distributions in Figure S3a-b are indicated in Figure S3c-d. We plot the spatial distribution of these fields normalized by the amplitude of the incident field in the vertical $y = 0$ plane. Finally, to obtain the collective coupling strength, we subtract the radiative contribution given by eq S46 to the scattered electric fields. The result of this subtraction is plotted in Figure 7b in the main text. We also subtract the radiative component of the magnetic fields following the same procedure. In the main text, we have used the $7 \times 7 \times 1$ grid in Figure S3a, but we have verified that the $15 \times 15 \times 1$ grid in Figure S3b yields almost identical coupling strength.

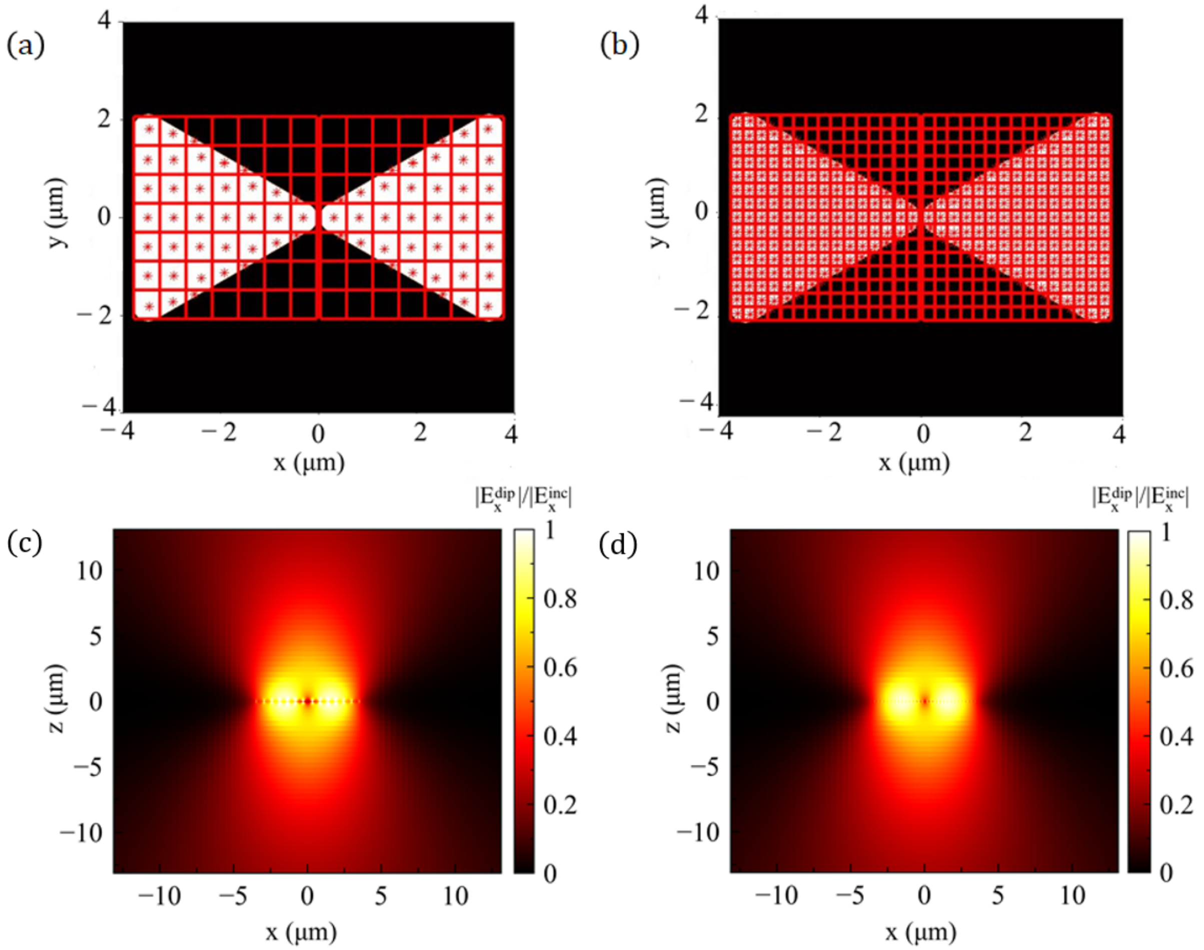

Figure S3: Illustration of the calculation of the far field radiation of a nanoantenna with nanogap distance of d_g = 60 nm. (a) Example of the 3D grid used for the calculation, plotted at $z = 0$ and corresponding to $7 \times 7 \times 1$ cells in each prism of the bowtie. Each cell has equal size, with their borders in the horizontal $x - y$ plane shown as red and horizontal lines. The cell size in the z direction corresponds to the thickness of the nanoantenna. The asterisks indicate the position of the dipoles inside the nanoantenna, while no dipoles are present in cells located entirely outside the nanoantenna. (b) Same as in (a) but using a 3D grid of $15 \times 15 \times 1$ cells for each prism. (c) Spatial distribution of the module of the x-component of the radiative contribution (far field radiation) of the electric field obtained by using eq S46 and the 3D grid of (a). The plotted field is normalized by the amplitude of the incident field, and the colors are saturated for values larger than 1. The coordinate axes are defined as in Figure 5a, with the center of the nanogap at $x = y = z = 0$. (d) Same that in (c) but using a 3D grid of $15 \times 15 \times 1$ cells.

S6 Further analysis of the semi-analytical expression developed for weakly radiative nanoantennas when analyzing plasmonic nanoantennas

In Figure 6b of the main text, we analyze the coupling strength for a cubic distribution of molecules and find a disagreement between i) the value g_{sa} calculated using the semi-analytical eq 12 before applying the correction for strong radiation and ii) the value g_{ho} obtained by fitting the spectra with the coupled harmonic oscillator model. However, in this figure, we did not consider the correction of g_{ho} given by eq 19, which can be necessary for strongly radiative systems. We show in Figure S4 that when using the corrected fit values $\tilde{g}_{ho}$ (dots in Figure S4), the results still disagree with the uncorrected semi-analytical results (blue solid line). This confirms the need of the improved semi-analytical treatment discussed in Section 3.7.

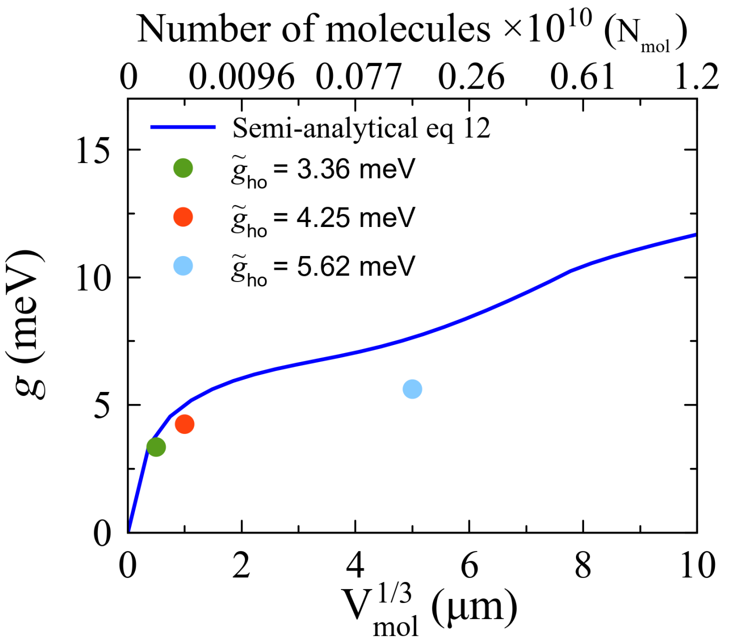

Figure S4: Comparison of the Au nanoantenna-molecules collective coupling strength obtained from the fit after adequate correction and from the semi-analytical treatment developed for weakly radiative nanoantennas. Blue line: Collective coupling strength g_{sa} obtained with the semi-analytical eq 12 in the main text without applying the correction for strong radiation introduced in section 3.7 of the main text. g_{sa} is plotted as a function of the side of the cubic volume occupied by the molecules $V_{mol}^{1/3}$ (bottom axis) and the number of molecules N_{mol} (upper axis). These results correspond to those plotted in Figure 6b in the main text. Dots: Corrected values of the collective coupling strength $\tilde{g}_{ho}$ obtained from the fit of the simulations to the equations derived with the coupled harmonic oscillator model, after applying eq 19. These results correspond to those plotted in Figure 7d in the main text. The green, red, and light blue dots correspond to cubic regions of volume $V_{mol} = 0.125$ μm^3, $V_{mol} = 1$ μm^3, and $V_{mol} = 125$ μm^3, respectively.

S7 Systematic analysis of the collective coupling strength of a bowtie nanoantenna with corner radius $r = 30$ nm

In sections 3.5-3.7 of the main text, we analyze the coupling of the molecules with a gold bowtie nanoantenna. In those simulations, the radius of the corners is $r = 300$ nm so that the shape of the SiC and Au nanoantennas (in the horizontal xy plane) is similar. For reference, we present in this section simulations of the gold nanoantennas with $r = 30$ nm, corresponding to the radius used for the SiC nanoantennas. The other dimensions of the

gold nanoantenna change only slightly: length 3.68 μm, width 4.18 μm, and 75 nm thickness, with nanopap distance $d_g = 60$ nm.

We compare in Figure S5 the collective coupling strength g_{sa} obtained for the gold nanoantennas with this radius $r = 30$ nm (red dashed line) and with the $r = 300$ nm value in the main text (blue solid line). The results are obtained for a cubic distribution of molecules, using the analytical eq 12 (with $\tilde{V}^{eff}_{rdc}$ obtained with the improved methodology in Section 3.7 in the main text), and are calculated as a function of the cubic root of the volume occupied by the molecules ($V^{1/3}_{mol}$, bottom axis), or, equivalently, of the number of molecules (N_{mol}, top axis). The general trend is similar in both cases, but the nanoantenna with $r = 30$ nm results in significantly larger coupling strength for relatively small V_{mol}, which can be attributed to larger field localization (smaller $|\tilde{V}^{eff}_{rdc}|$). On the other hand, as the volume occupied by the molecules increases, the coupling strength becomes more weakly dependent on the field localization (i.e. on the radius r), as discussed in the text. We attribute most of the difference in coupling strength that remains when the nanoantennas are completely surrounded by molecules to the shift of the resonant frequency with r, which changes the ratio of energy inside and outside (as we are not in the quasistatic regime, the modification of the nanoatenna shape could also introduce an additional change on this ratio, beyond that introduced by the change in resonant frequency).

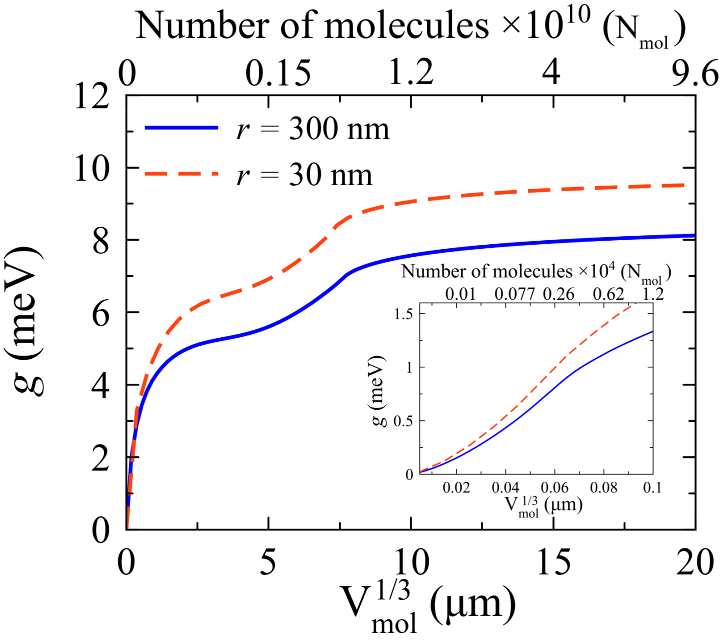

Figure S5: Comparison of the collective coupling strength for different values of the radius r of the corners of the Au plasmonic nanoantenna. The blue solid line corresponds to the results shown in Figure 7(d) for a Au nanoantenna with $r = 300$ nm, and the red dashed line corresponds to the results for $r = 30$ nm, both calculated through numerical integration of eq 12 of the main text after applying the correction for strong radiation in section 3.7 of the main text. The collective coupling strength is plotted as a function of the cubic root of the cubic volume occupied by the molecules $V_{mol}^{1/3}$ (bottom axis) and the corresponding number of molecules N_{mol} (upper axis). The nanogap distance is $d_g = 60$ nm in both cases.

References

(1) Barra-Burillo, M.; Muniain, U.; Catalano, S.; Casanova, F.; Hueso, L. E.; Aizpurua, J.; Esteban, R.; Hillenbrand, R.; others Microcavity phonon polaritons from the weak to the ultrastrong phonon–photon coupling regime. Nature communications **2021**, 12, 1–9.

(2) González-Tudela, A.; Huidobro, P. A.; Martín-Moreno, L.; Tejedor, C.; García-Vidal, F. J. Theory of Strong Coupling between Quantum Emitters and Propagating Surface Plasmons. Physical Review Letters **2013**, 110, 126801.

(3) Abujetas, D. R.; Feist, J.; García-Vidal, F. J.; Rivas, J. G.; Sánchez-Gil, J. A. Strong coupling between weakly guided semiconductor nanowire modes and an organic dye. Physical Review B **2019**, 99, 205409.

(4) Canales, A.; Baranov, D. G.; Antosiewicz, T. J.; Shegai, T. Abundance of cavity-free polaritonic states in resonant materials and nanostructures. The Journal of Chemical Physics **2021**, 154.

(5) Dayal, G.; Morichika, I.; Ashihara, S. Vibrational strong coupling in subwavelength nanogap patch antenna at the single resonator level. The Journal of Physical Chemistry Letters **2021**, 12, 3171–3175.

(6) Crotti, G.; Schirato, A.; Zaccaria, R. P.; Della Valle, G. On the limits of quasi-static theory in plasmonic nanostructures. Journal of Optics **2021**, 24, 015001.

(7) Muniain, U.; Aizpurua, J.; Hillenbrand, R.; Martín-Moreno, L.; Esteban, R. Description of ultrastrong light-matter interaction through coupled harmonic oscillator models and their connection with cavity-QED Hamiltonians. Nanophotonics **2024**,

(8) Fox, A. M. Quantum optics: an introduction; Oxford university press, 2006; Vol. 15.

(9) Ribeiro, S.; Aizpurua, J.; Esteban, R. Influence of direct dipole-dipole interactions on the optical response of two-dimensional materials in strongly inhomogeneous infrared cavity fields. Physical Review A **2023**, 108, 043718.

(10) Novotny, L.; Hecht, B. Principles of Nano-Optics; Cambridge University Press, 2006.

(11) Gerry, C. C.; Knight, P. L. Introductory quantum optics; Cambridge university press, 2023.

(12) Ruppin, R. Electromagnetic energy density in a dispersive and absorptive material. Physics Letters A **2002**, 299, 309–312.

(13) Maier, S. A. Plasmonic field enhancement and SERS in the effective mode volume picture. Optics Express **2006**, 14, 1957–1964.

(14) Loudon, R. The propagation of electromagnetic energy through an absorbing dielectric. Journal of Physics A: General Physics **1970**, 3, 233.

(15) Wang, F.; Shen, Y. R. General Properties of Local Plasmons in Metal Nanostructures. Physical Review Letters **2006**, 97, 206806.

(16) Jackson, J. D. Classical electrodynamics. 1999.
